# Magdalenian and Epimagdalenian chronology and palaeoenvironments at Kůlna Cave, Moravia, Czech Republic

**DOI:** 10.1007/s12520-020-01254-4

**Published:** 2020-12-17

**Authors:** Hazel Reade, Sonja B. Grimm, Jennifer A. Tripp, Petr Neruda, Zdeňka Nerudová, Martina Roblíčková, Kerry L. Sayle, Rebecca Kearney, Samantha Brown, Katerina Douka, Thomas F. G. Higham, Rhiannon E. Stevens

**Affiliations:** 1 Institute of Archaeology, University College London, London, UK; 2 Centre for Baltic and Scandinavian Archaeology (ZBSA), Foundation Schleswig-Holsteinian State Museums Schloss Gottorf, Schleswig, Germany; 3 Department of Chemistry, University of San Francisco, San Francisco, USA; 4 Anthropos Institute, Moravian Museum, Brno, Czech Republic; 5 Centre for Cultural Anthropology, Moravian Museum, Brno, Czech Republic; 6 Scottish Universities Environmental Research Centre, East Kilbride, UK; 7 Department of Archaeology, Max Planck Institute for the Science of Human History, Jena, Germany; 8 Research Laboratory for Archaeology and the History of Art, University of Oxford, Oxford, UK

**Keywords:** Sulphur isotopes, Nitrogen isotopes, Carbon isotopes, Final Palaeolithic, Late Upper Palaeolithic, Permafrost

## Abstract

Kůlna Cave is the only site in Moravia, Czech Republic, from which large assemblages of both Magdalenian and Epimagdalenian archaeological materials have been excavated from relatively secure stratified deposits. The site therefore offers the unrivalled opportunity to explore the relationship between these two archaeological phases. In this study, we undertake radiocarbon, stable isotope (carbon, nitrogen and sulphur), and ZooMS analysis of the archaeological faunal assemblage to explore the chronological and environmental context of the Magdalenian and Epimagdalenian deposits. Our results show that the Magdalenian and Epimagdalenian deposits can be understood as discrete units from one another, dating to the Late Glacial between c. 15,630 cal. BP and 14,610 cal. BP, and c. 14,140 cal. BP and 12,680 cal. BP, respectively. Stable isotope results (δ^13^C, δ^15^N, δ^34^S) indicate that Magdalenian and Epimagdalenian activity at Kůlna Cave occurred in very different environmental settings. Magdalenian occupation took place within a nutrient-poor landscape that was experiencing rapid changes to environmental moisture, potentially linked to permafrost thaw. In contrast, Epimagdalenian occupation occurred in a relatively stable, temperate environment composed of a mosaic of woodland and grassland habitats. The potential chronological gap between the two phases, and their associations with very different environmental conditions, calls into question whether the Epimagdalenian should be seen as a local, gradual development of the Magdalenian. It also raises the question of whether the gap in occupation at Kůlna Cave could represent a change in settlement dynamics and/or behavioural adaptations to changing environmental conditions.

**Supplementary Information:**

The online version contains supplementary material available at 10.1007/s12520-020-01254-4.

## Introduction

Kůlna Cave, situated in Moravia, Czech Republic, is widely considered one of the most important Palaeolithic archaeological sites in Central Europe, owing to its deep stratigraphy, multiple occupation horizons, and large lithic and faunal assemblages dating from Marine Isotope Stage (MIS) 6 to the Holocene (Valoch 1988; Svoboda 2005; Nerudová and Neruda 2014; Neruda and Nerudová 2014). It is one of very few Moravian sites to contain both Late Upper Palaeolithic Magdalenian and Final Palaeolithic Epimagdalenian assemblages in relatively secure stratified deposits (Valoch 1988; Neruda and Valoch 2007). It therefore offers a rare opportunity to explore the chronological and environmental context of, and relationship between, these two archaeological phases at the same location (Valoch 1988; Neruda and Valoch 2007). Through radiocarbon dating and stable isotope analysis (δ^13^C, δ^15^N, δ^34^S) of the archaeological faunal assemblage, we investigate palaeoenvironmental conditions during Magdalenian and Epimagdalenian phases of human occupation at Kůlna Cave. These post-Last Glacial Maximum (LGM) assemblages date to the Last Glacial Termination and Late Glacial Interstadial in Central Europe, which correspond approximately to, but are not synonymous with, Greenland Stadial 2.1a (GS-2.1a) and Greenland Interstadial 1 (GI-1), and the Oldest Dryas and Bølling-Allerød, respectively (Rasmussen et al. 2014; Vočadlová et al. 2015; Kuneš and Abraham 2017). Thus, this study enables the Magdalenian and Epimagdalenian occupation of the wider Moravian landscape to be considered against the backdrop of post-LGM environmental change.

While some areas of Central Europe were largely abandoned by human groups during the LGM (c. 24,000 to 19,000 BP, Hughes et al. 2015), human presence, associated with the Epigravettian, appears to have persisted in Moravia at least intermittently (Nerudová and Neruda 2015; Nerudová et al. 2016). As such, the post-LGM expansion of the Magdalenian (c. 17,000 to 15,000 BP) may not have been into an unoccupied landscape, as it was in other regions (Nerudová 2010; Maier 2015; Nerudová and Neruda 2015, Maier et al. 2020). Magdalenian presence in Moravia has been broadly correlated with cold, periglacial climates associated with the Last Glacial Termination (GS-2.1a), although a mosaic of habitats existed in the region (Nerudová et al. 2016). This contrasts to the Final Palaeolithic (termed the Late Palaeolithic in Central Europe), which includes the Epimagdalenian. This phase is characterised by a greater diversity of lithic industries and is typically associated with more temperate environments of the Late Glacial Interstadial (GI-1) (Svobodová 1988; Valoch 1996; Nerudová et al. 2014; Moník and Pankowská 2020). Against this backdrop of environmental and archaeological change, genetic data suggests significant population turnover and migration may have occurred within Europe across this time period (Fu et al. 2016; Posth et al. 2016). Combined, this evidence has led to much debate about whether certain Late Upper and Final Palaeolithic technocomplexes were local developments of preceding industries or non-local imports, and whether environmental and ecological change driven by post-LGM climate variability, were factors in such developments (e.g. Valoch 1988, 2010; Jochim et al. 1999; Vencl 1999; Blockley et al. 2006; Verpoorte 2009; Moník, 2014). In Moravia, the Epimagdalenian has typically been viewed as a local development of the Magdalenian (Valoch 1988; Moník 2014). However, uncertainties over the chronological relationship between the two cultures and the duration of each (Mook 1988; Nerudová and Neruda 2014; Moník 2014) make drawing direct inferences about the environmental conditions in which they operated challenging. Kůlna Cave, with its Magdalenian and Epimagdalenian assemblages, offers an unrivalled opportunity to examine this topic further and develop improved understandings of Magdalenian and Epimagdalenian occupation in the wider Moravian landscape. By focusing analysis on the zooarchaeological assemblages from the site, palaeoenvironmental inferences directly associated to phases of human presence in the region can be made.

## Background

**Fig. 1:**
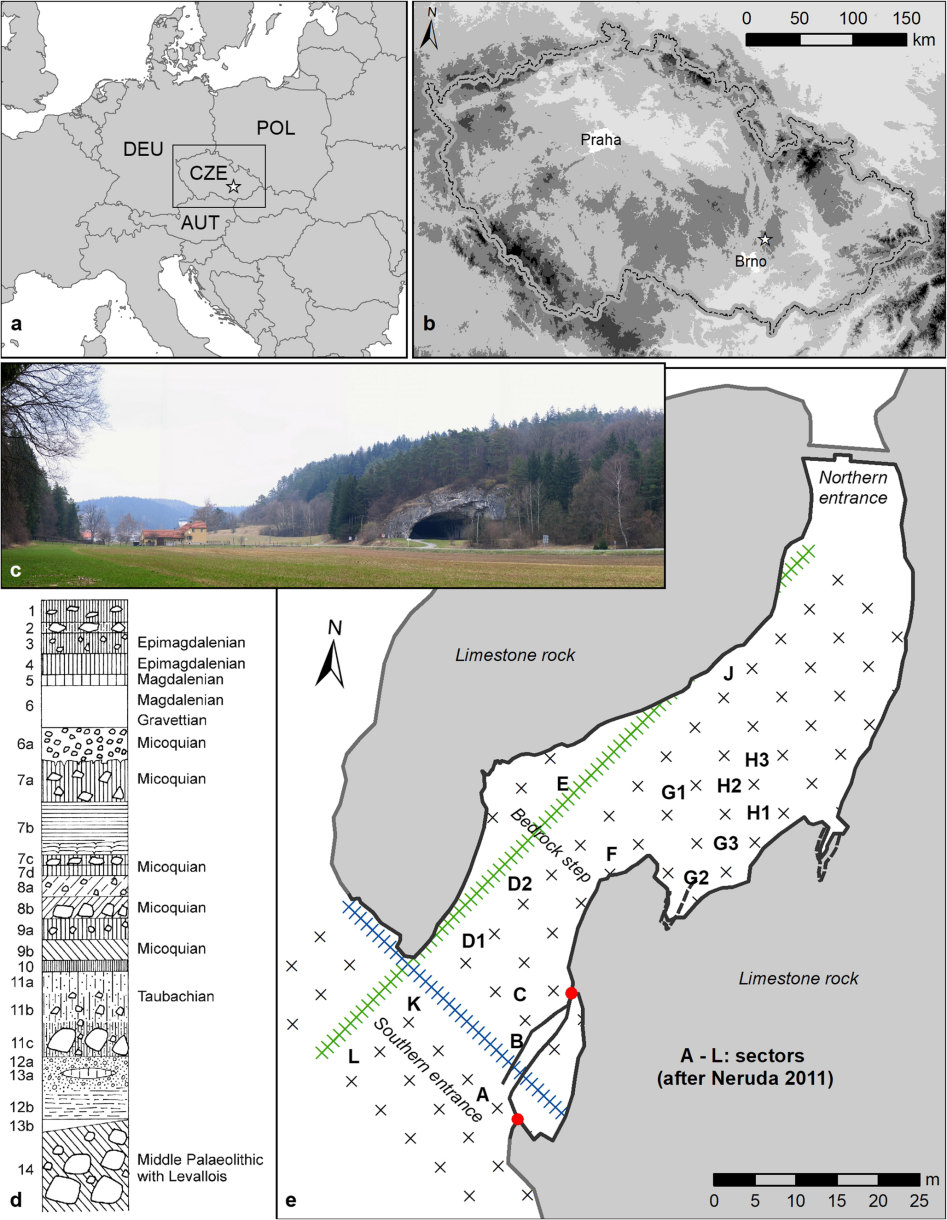
Location of Kůlna Cave in the Czech Republic marked by the white star symbol (**a** and **b**); position of Kůlna Cave in the Punkva River valley showing the southern entrance to the cave (**c**); schematic diagram of the sedimentological and archaeological horizons identified in the cave sediments (**d**); plan of the excavation sectors defined during the Valoch excavations conducted between 1961 and 1976 (**e**)

Kůlna Cave (49° 24‵ 26‶, 16° 44‵ 16‶ E, Fig. 1a–b) is located in the Punkva River valley (Fig. 1c) at approximately 470 m asl (Nerudová et al. 2014). Situated at the northern margins of the Moravian Karst, the site is in close proximity to a varied landscape consisting of wide valleys, narrow gorges and limestone plateaus (Neruda and Valoch 2007). The most extensive excavations at the site were conducted between 1961 and 1976 (Valoch 1988). These excavations revealed a 15-m deep sequence of 14 sedimentological horizons (Fig. 1d; Valoch 1988). Layers 14 to 6a were assigned to the Middle Palaeolithic (Mousterian with Levallois method, Taubachian, and Micoquian) and Layers 6 to 3 to the Upper Palaeolithic (Gravettian, Magdalenian, Epimagdalenian) and Mesolithic (Valoch 1988). The possible presence of an Epigravettian phase at the site has been suggested but recent analysis has discounted this (Nerudová and Moník 2019). In this study, we focus on the layers which contain evidence of Magdalenian (Layer 6 and 5) and Epimagdalenian (Layer 4) activity. Epimagdalenian lithics were also found in Layer 3 but were admixed with Holocene-aged Mesolithic material. Thus, to avoid the potential inclusion of Holocene-aged samples, Layer 3 was not included in our analysis.

Previous analysis of the post-LGM Kůlna Cave lithic assemblage inferred a gradual development of the Epimagdalenian from the Magdalenian industry across Layer 6 to Layer 3 (Valoch 1988). However, more recent analysis has described the Layer 6 and 5 lithics as being technologically uniform across the two layers (Nerudová and Moník 2019), suggesting the interpretation of a gradual development should be questioned. The stratigraphy of the post-LGM sediments is complex, and preservation and depth of layers varied considerably between different excavation areas within the cave (Fig. 1e). Admixture of the post-LGM archaeological horizons, particularly Layer 5, is evident in the radiocarbon dates already obtained on the fauna from this layer (Table 1; Mook 1988; Nerudová and Neruda 2014), further calling into question the integrity of the implied Layer 6 to 3 sequence of lithic development. Despite these complexities, pre-existing chronological data from Layer 6 to 4 attest to a series of episodic post-LGM human phases at the site between c. 15,250 cal. BP and c. 12,780 cal. BP (Nerudová and Neruda 2014). This time interval spans the final part of GS-2.1a (before c. 14,650 cal. BP) and the whole of the GI-1 (c. 14,650 cal. BP to c. 12,850 cal. BP), representing a period of considerable environmental change.

**Table 1:** Radiocarbon determinations from Layers 4–6 at Kůlna Cave. Calibrated age range is reported at 2 sigma uncertainty on the IntCal20 timeline. Reported δ^13^C isotope values were measured by IRMS as part of the AMS dating process. References: (1) this study, (2) Mook, 1988, (3) Nerudová, and Neruda, 2014. *n.d*. no data. Units in brackets correspond to the final system of unit division of the cave

UPNORTH project sample code	Sample type	Species	Element	Layer	Sector	Unit	δ^13^C	C/N atomic ratio	Lab code	^14^C date	Dating method	Calibrated Age BP (2σ)	Ref
n/a	bone	n.d	n.d	4	K	III-IV/F	n.d	n.d	GrN-6102	11,470 ± 105	conv.	13,162–13,576	2
n/a	charcoal	n.d	n.d	4	B/C	I-II/L,M,P,R	n.d	n.d	GrN-11051	2,135 ± 45	conv.	1,992–2,305	2
UPN-162	bone	*A. alces*	metatarsal	4	A	7f = P/VII	− 21.2	n.d	OxA-25284	11,820 ± 50	AMS	13,520–13,790	3
UPN-163	bone	*A. alces*	metatarsal	4	A	1a = T/XIII	− 20.7	n.d	OxA-25285	11,770 ± 55	AMS	13,501–13,765	3
UPN-164	bone	*Equus* sp.	tibia	4	A	6f = P/VIII	− 19.9	n.d	OxA-25286	11,070 ± 50	AMS	12,846–13,096	3
UPN-120	bone	*Equus* sp.	phalange 2	5	A	6 g (O/8)	− 21.1	3.4	OxA-V-2777-55C	12,810 ± 60	AMS	15,110–15,535	1
UPN-128	bone	*Equus* sp.	metacarpal	5	D	S/III,IV	− 21.2	3.3	OxA-V-2777-56C	11,510 ± 50	AMS	13,298–13,490	1
n/a	bone	n.d	n.d	5	C	I-III/K-L	n.d	n.d	GrN-6103	17,480 ± 155	conv.	20,750–21,733	2
UPN-165	bone	uncertain	unknown	5	C	I/L,M	− 20.1	n.d	OxA-25287	11,010 ± 50	AMS	12,783–13,084	3
UPN-166	bone	*Equus* sp.	unknown	5	C	I-III/K,L	− 20.4	n.d	OxA-25288	12,600 ± 60	AMS	14,565–15,215	3
UPN-102	bone	*Equus* sp.	phalange	6	A	6a (T/VIII)	− 21.5	3.3	OxA-V-2775-57C	12,910 ± 60	AMS	15,242–15,634	1
UPN-171	bone	*R. tarandus*	humerus	6	G1	38/L	− 19.4	3.3	OxA-V-2793-53C	12,650 ± 50	AMS	14,925–15,254	1
UPN-171	bone	*R. tarandus*	humerus	6	G1	38/L	− 19.4	n.d	OxA-25291	12,620 ± 60	AMS	14,611–15,248	3
n/a	charcoal	n.d	n.d	6	G2	37-38/M,O	n.d	n.d	GrN-5097	11,590 ± 80	conv.	13,305–13,600	2
n/a	charcoal	n.d	n.d	6	D2/C	13-14/I-L	n.d	n.d	GrN-11053	11,450 ± 90	conv.	13,166–13,490	2
n/a	charcoal	n.d	n.d	6	D2/C	13-14/I-L	n.d	n.d	GrN-11052	7,550 ± 110	conv.	8,048–8,590	2
UPN-169	bone	*Equus* sp.	pelvis	6	G1	37-38/M	− 20.8	n.d	OxA-25289	12,575 ± 60	AMS	14,516–15,179	3
UPN-170	bone	*Equus* sp.	tibia	6	G1	36-37/N	− 20.9	n.d	OxA-25290	12,555 ± 60	AMS	14,467–15,156	3

The earliest confirmed Magdalenian site in Moravia (Balcarka Cave) dates to c. 17,000 cal. BP and corresponds to a period of initial temperature increase after the LGM (Nerudová 2010). The Magdalenian phases at Kůlna Cave date to a later period, starting after c. 15,600 cal. BP, when a more sustained Magdalenian presence is evident in the region (Valoch and Neruda 2005; Nerudová and Neruda 2014). Mean annual air temperature for this time period (c. 18,000–15,000 cal. BP) ranged from 6 to 11 °C, estimated from tooth enamel δ^18^O values (Kovács et al. 2012). While most regional pollen spectra indicate a largely treeless landscape dominated by herb and grass taxa, localised pockets of open birch and willow woodland also existed, suggesting the presence also of more temperate microclimates (Svobodová 1988; Pokorný 2002; Hošek et al. 2014; Nerudová et al. 2016). At Kůlna Cave reindeer, hare and horse are most common in the Layer 6 (Magdalenian) faunal assemblage, with mammoth, bear, bovids, woolly rhino, arctic fox and birds also being present (Valoch et al. 1969; Zelinková 1998). The occurrence of thermophilic species, albeit small in number, within a predominantly cold-adapted fauna has been interpreted as further evidence in support of the presence of warmer microenvironments in the region (Valoch et al. 1969; Zelinková 1998). The increased frequency of hare in Layer 6 has been interpreted as reflecting a prey preference of the Magdalenian hunters (Zelinková 1998). A similar mixture of cold-climate and thermophilic species also occurs in the Layer 5 (Magdalenian) faunal assemblage (Valoch et al. 1969). However, the relative proportions of these species differ considerably in different sectors of the cave, such that it has been suggested that the layer may contain intrusive material or represent an admixture of material of differing ages (Valoch et al. 1969).

The Epimagdalenian in Moravia appears to have taken place under considerably different environmental conditions to the Magdalenian. Dating to after c. 14,000 cal. BP, the Epimagdalenian occurred during a period of warmer, wetter climate when an increase in pine and birch woodland is evident in local pollen records (Pokorný and Jankovská 2000; Nerudová et al. 2014; Kadlec et al. 2015). Such environmental changes are reflected in the spectrum of exploited fauna, with red deer, elk, and large bovids becoming more common in the zooarchaeological assemblages of the region (Valoch 1988, 2001). At Kůlna Cave, the Layer 4 (Epimagdalenian) faunal assemblage has been described as a typical forest fauna, which also contains elements characteristic of colder environments (Valoch et al. 1969). Species include elk, red deer, aurochs, horse, reindeer, roe deer, red fox, Eurasian beaver, hare, birds, wild boar, and brown bear, along with mammoth and woolly rhino, which occur infrequently and are interpreted as being intrusive (Valoch et al. 1969). Unlike the Layer 6 Magdalenain assemblage, hunting preference in the Epimagdalenian appears to have been restricted to medium and large prey species (Valoch et al. 1969).

Therefore, while existing palaeoenvironmental and zooarchaeological data allows broad-scale characterisation of the landscapes and ecological contexts Magdalenian and Epimagdalenian populations inhabited, a number of questions remain. The potentially episodic nature of human activity in the region combined with the speed of landscape change at this time hinders detailed correlation between environmental conditions and the timing of human presence. Furthermore, while archaeological faunal assemblages provide insight into prey species availability and hunting preferences, the species composition of an assemblage may not directly correspond to the natural composition of species present in the landscape, and thus palaeoenvironmental interpretations from this archive may be biased. Stable isotope analysis (δ^13^C, δ^15^N, δ^34^S) and radiocarbon dating of hunted fauna provide important complementary evidence in this regard. Faunal isotopic compositions reflect the environment and ecology of the animals’ home range at the time the animal lived, and analysis of zooarchaeological material allows these records to be directly tied to periods when humans were active within the landscape. Faunal δ^13^C values are largely determined by dietary ecology but also respond to environmental variables such as vegetation density and type, temperature and water availability (Heaton 1999; Stevens and Hedges 2004; Drucker et al. 2008; Kohn 2010). Both faunal δ^15^N and δ^34^S values reflect underlying soil processes related to different aspects of the soil environment (e.g. temperature, nutrient status, microbial activity, water and oxygen content, underlying lithology (Thode 1991; Amundson et al. 2003; Craine et al. 2015a, b; Nehlich 2015; Nitsch et al. 2019)). Therefore, combined δ^13^C, δ^15^N, and δ^34^S analysis of archaeological fauna can be used to better understand the terrestrial environment in which people hunted.

## Material and methods

### Sample selection

Fauna from Layers 6, 5, and 4 were targeted for analysis. All analysed material comes from the Valoch excavations of the 1960s/70s for which relatively good stratigraphic information is available in the archival records of the Moravian Museum, Brno, in K. Valoch’s original field notebooks, and in publications relating to the excavations (Valoch 1969, 1988). Elk (*Alces alces*), aurochs (*Bos primigenius*), red deer (*Cervus elaphus*), horse (*Equus* sp.), and reindeer (*Rangifer tarandus*) were selected for analysis as they represent the most common prey fauna at the site (Valoch 1988). One saiga antelope (*Saiga tatarica*) specimen was also analysed. This species is relatively rare in the Kůlna Cave faunal assemblage and has very specific environmental requirements comprising of flat, arid steppe with < 20 cm snowfall (Bannikov et al. 1961). Samples come from sectors A, C, D, and G1 (Fig. 1e), representing locations both within the main cavern and at the main cave entrance. One hundred and one faunal specimens were sampled for stable isotope analysis. Eight of these samples had been previously radiocarbon dated (Nerudová and Neruda 2014), 1 was re-dated, and a further 3 were dated as part of this study. A full list of samples is provided in Supplementary Information 1.

### Sample preparation

A small sample of bone (0.45–1.57 g) was collected from each specimen using a dental drill with either a small cutting wheel or tungsten burr attachment. Collagen extraction was performed at University College London (UCL) using a modified version of the Oxford Radiocarbon Accelerator Unit (ORAU) collagen extraction procedures (AF and AG methods; Brock et al. 2010), which is based on a modified version of the Longin (1971) method. Samples were demineralised in 0.5 M hydrochloric acid (HCl) at 4 °C and then thoroughly rinsed with ultrapure water. Samples were then gelatinised in pH 3 HCl solution at 75 °C for 48 h and filtered using a pre-cleaned Ezee-filter. For some samples, including all those to be radiocarbon dated, the filtrate was then passed through a pre-cleaned 15–30 kD ultrafilter, with the > 30 kD fraction collected and freeze-dried (AF method). For other samples, the ultrafiltration step was omitted (AG method); while ultrafiltration has been shown to successfully improve the removal of contaminants that can influence radiocarbon determinations (Higham et al. 2006), it has been shown to be unnecessary for stable isotope analysis (Sealy et al. 2014; Szpak et al. 2017). Of the 101 samples processed, 99 had collagen yields adequate for subsequent analysis. Details of pretreatment methodology and yield data for each sample are given in the Supplementary Information 1.

### Radiocarbon analysis

In total, four samples were radiocarbon dated in this study. One of these samples (Layer 6, Sector G1, UPN-171) had previously been radiocarbon dated (OxA-25291) and was re-dated to check quality and reproducibility between our analysis and previous work. The remaining three samples provide new chronological information from the site from Layer 6 (Sector A, UPN-102) and 5 (Sector A and D, UPN-120 and UPN-128). Radiocarbon dating was performed on extracted collagen at ORAU using their standard procedures (Brock et al. 2010). Approximately 5 mg of dry collagen per sample was weighed into pre-baked tin capsules and combusted using an elemental analyser coupled to an isotope ratio mass spectrometer, employing a splitter to allow for collection of the CO_2_ (Bronk Ramsey and Humm 2000; Brock et al. 2010). Samples were graphitised by reduction of collected CO_2_ over an iron catalyst in an excess H_2_ atmosphere at 560 °C (Bronk Ramsey and Hedges 1997; Dee and Bronk Ramsey 2000). The ^14^C dates were measured on the Oxford AMS system using a caesium ion source for ionisation of the solid graphite sample (Bronk Ramsey et al. 2004). To denote the bone pretreatment at UCL rather than at ORAU, all measured dates were given “OxA-V-wwww-pp” numbers, where “wwww” indicates the wheel number, and “pp” is the position of the sample on the wheel (Brock et al. 2010). Background corrections were applied to our dates to account for the collagen extraction being performed at UCL, following the method outlined by Wood et al. (2010). A full description of our correction methodology is detailed in Reade et al. (2020a). Corrected dates are denoted by adding a “C” to the end of the date code assigned by ORAU. Uncorrected measured date values as well as further details of the correction calculations are provided in the Supplemental Information 2. Results are reported as uncalibrated radiocarbon dates (^14^C BP) and discussed as calibrated dates BP (cal. BP). Date calibration was performed using OxCal 4.4 (Bronk Ramsey 2020) and the IntCal20 dataset (Reimer et al. 2020).

### Stable isotope analysis

Isotopic compositions were determined on the extracted bone collagen using a Delta V Advantage continuous-flow isotope ratio mass spectrometer coupled via a ConfloIV to an IsoLink Elemental Analyser (Thermo Scientific, Bremen) at the Scottish Universities Environmental Research Centre. Samples were weighed into tin capsules (~ 1.2–1.5 mg) and combusted in the presence of oxygen in a single reactor containing tungstic oxide and copper wires at 1020 °C to produce N_2_, CO_2_, and SO_2_. A magnesium perchlorate trap was used to eliminate water produced during the combustion process, and the gases were separated in a GC column heated between 70 °C and 240 °C. Helium was used as a carrier gas throughout the procedure. N_2_, CO_2_, and SO_2_ entered the mass spectrometer via an open split arrangement within the ConfloIV and were analysed against their corresponding reference gases. For every ten archaeological samples, three in-house standards that are calibrated to the International Atomic Energy Agency (IAEA) reference materials USGS40 (L-glutamic acid, δ^13^C_VPDB_ = – 26.4‰, δ^15^N_AIR_ = – 4.5‰), USGS41 (L-glutamic acid, δ^13^C_VPDB_ = + 37.6‰, δ^15^N_AIR_ = – 47.6‰), IAEA-CH-6 (sucrose, δ^13^C_VPDB_ = – 10.5‰), IAEA-N-1 (ammonium sulphate, δ^15^N_AIR_ = + 0.4‰), IAEA-S-1 (silver sulphide, δ^34^S_VCTD_ = – 0.3‰), IAEA-S-2 (silver sulphide, δ^34^S_VCTD_ = 22.7‰), IAEA-SO-5 (barium sulphate, δ^34^S_VCTD_ = 0.5‰), and IAEA-SO-6 (barium sulphate, δ^34^S_VCTD_ = – 34.1‰) were run (Sayle et al. 2019). Results are reported as per mil (‰) relative to the internationally accepted standards VPDB, AIR, and VCDT. Precision was determined to be ± 0.1‰ for δ^13^C, ± 0.2‰ for δ^15^N, and ± 0.3‰ for δ^34^S on the basis of repeated measurements of calibration standards.

### ZooMS analysis

Peptide mass fingerprinting, specifically ZooMS (Zooarchaeology by Mass Spectrometry; Buckley et al. 2009), was performed on 7 samples where taxonomic classification was uncertain. These included two previously dated but unidentified bone fragments from Layer 5 that bore evidence of anthropogenic action but were not identifiable to species by macroscopic zooarchaeological methods (Nerudová and Neruda 2014). A further five samples where the distinction between reindeer or red deer could not be ascertained by macroscopic zooarchaeological analysis were also analysed by ZooMS. Full details of the ZooMS methodology and results are provided in the Supplementary Information 3.

## Results

### Radiocarbon dating

**Fig. 2:**
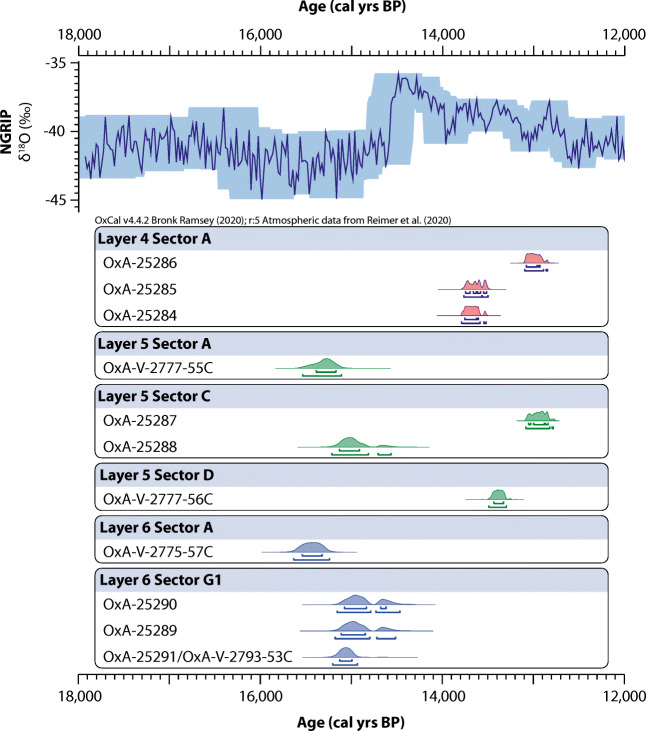
Calibrated ultrafiltered AMS radiocarbon dates from Kůlna Cave. Calibration performed using OxCal 4.4 (Bronk Ramsey 2020) and the INTCAL20 dataset (Reimer et al. 2020) and shown against the NGRIP δ^18^O record (Andersen et al. 2006; Svensson et al. 2006). The shaded area surrounding the NGRIP δ^18^O values indicates age uncertainty at 2 sigma, roughly equivalent to the maximum ice layer counting error. The GICC05 (ice core) and IntCal20 timescales were aligned using the INTIMATE chronological database integration tool (Bronk Ramsey et al. 2014, 2019) and the time-transfer function devised by Adolphi and Muscheler (2016), which is still applicable between IntCal20 and GICC05 timescales (Muscheler et al. 2020). OxA-25284 to OxA-25291 dates are from Nerudová and Neruda (2014), OxA-V- dates are previously unpublished

Four radiocarbon dates were made on samples from Layers 6 and 5 (Table 1, Fig. 2), which provide additional chronological data to that offered by previous studies (Mook 1988; Nerudová and Neruda 2014). UPN-171 (Layer 6, Sector G1), which had previously been dated, produced a date of 12,650 ± 50 ^14^C BP (OxA-V-2793-53C). This is statistically indistinguishable (Chi-square test *x*^*2*^ = 0.1, *p* > .05) from the previous date of 12,620 ± 60 ^14^C BP (OxA-25291; Nerudová and Neruda 2014) and demonstrates the reproducibility between our analysis and previous work. An error-weighted mean value of 12,638 ± 39 ^14^C BP will be used for this sample in subsequent discussions. The newly analysed sample from Layer 6 (UPN-102) produced a date of 12,910 ± 60 ^14^C BP (OxA-V-277-57C). The sample was selected for dating to provide an age from Sector A, an area of the cave that had been identified as containing a dense accumulation of Magdalenian artefacts (Blinková and Neruda 2015). All AMS-dated Layer 6 material from this study and that of Nerudová and Neruda (2014) correspond to the latter part of GS-2.1a (i.e. before the interstadial warm period). This contrasts to three conventional radiocarbon determinations from Layer 6 made on charcoal samples (Mook 1988), which date to GI-1 and the early Holocene (Table 1). However, these conventional dates are now considered unreliable based on an improved understanding of stratigraphic provenance and post-depositional mixing (Nerudová and Neruda 2014). Therefore, the Layer 6 data suggests human activity at the cave most likely took place between c. 15,630 cal. BP and 14,610 cal. BP (Bayesian statistical model output (1σ) rounded to the nearest 10, based on a single-phase input for the Magdalenian (Supplementary Information 4)). However, the Sector A date is around 300 years older than those from Sector G1, likely indicating that more than one phase of activity took place at the site during GS-2.1a. This finding supports the interpretation of the lithic assemblage indicating repeat visits to the cave during the Magdalenian (Blinková and Neruda 2015; Nerudová and Moník 2019).

The two newly analysed samples from Layer 5 produced dates of 12,810 ± 60 ^14^C BP (OxA-V-277-55C, Sector A, UPN-120) and 11,510 ± 50 ^14^C BP (OxA-V-277-56C, Sector D, UPN-128). These dates provide further evidence of mixing in the faunal assemblage in this layer and, importantly, indicate that stratigraphic mixing does not appear to be restricted to one particular sector within the cave. It is noteworthy that the Layer 5 Sector A date is most similar in age to the Layer 6 Sector A date (Fig. 2) demonstrating the importance of considering both the sector and layer when interpreting the data. Rather than the Layer 5 deposit representing an extended period of accumulation, results suggest the assemblage contains material from two distinct and disparate time periods that can be correlated with the phases of occupation represented within the Layer 6 and Layer 4 sediments (Fig. 2). The available radiocarbon dates suggest that Layers 6 and 4, which are archaeologically and sedimentologically different (Valoch 1988), could be separated by a hiatus in site use of c. 400–1000 years. The latter phase of human use of the cave appears to have taken place between c. 14,140 cal. BP and 12,680 cal. BP (Bayesian statistical model output (1σ) rounded to the nearest 10, based on a single-phase input (Supplementary Information 4)), broadly correlating to the latter part of GI-1 (roughly GI-1c-a). As with Layer 6, the GI-1 dates from Layer 5/4 unlikely represent a single occupation event.

### Isotope and ZooMS results

All samples (*n* = 99) analysed for collagen δ^13^C, δ^15^N, and δ^34^S had C/N ratios (3.1–3.4) indicative of well-preserved collagen for carbon and nitrogen isotope analysis, while 91 samples had C/S and N/S ratios (315–885 and 100–300) within the quality range determined for sulphur isotope analysis (Ambrose 1990; DeNiro 1985; Nehlich and Richards 2009). All data are reported in Supplementary Information 1. Only data that met the quality criteria are included in the following discussion of data.

ZooMS analysis established one of the two previously dated and unidentified samples from Layer 5 (OxA-25288/UPN-166) as belonging to horse. The other (OxA-25287/UPN-165) was not discernible to genus but is identified as most likely being elk or red deer. A further 5 Cervid samples were analysed by ZooMS where attribution to red deer or reindeer was uncertain. ZooMS analysis is able to distinguish reindeer from red deer but is unable to reliably differentiate between red deer and other Cervids such as elk and fallow deer (*Dama dama*) (Buckley et al. 2009; Buckley and Collins 2011). ZooMS results from the Cervid samples from Layer 6 (UPN-096 and UPN-147) excluded reindeer as a possible identification. For the Layer 4 samples, one (UPN-085) was confirmed and two (UPN-053 and UPN-060) were excluded as reindeer. For samples that are not reindeer, the most likely species attribution is red deer or elk based on contextual information combined with macroscopic zooarchaeological, ZooMS, and stable isotope analysis. Full results and a further discussion of the data are provided in the Supplementary Information 3.

For the stable isotope results, samples where species identification continues to be uncertain after both macroscopic zooarchaeological and ZooMS analysis (Supplementary Information 3) are indicated in appropriate figures but are excluded from inter-species statistical comparisons. Two further Cervidae Layer 5 samples (UPN-107 and UPN-115) were also excluded from subsequent data analysis due to questions over their identification based on their isotope results. UPN-107 was originally identified by macroscopic zooarchaeological analysis as reindeer and not subjected to ZooMS analysis. However, its isotopic results fall > 1.5 times outside the interquartile range of all reindeer δ^13^C and δ^15^N results. As discussed in the following paragraphs, its isotopic values (δ^13^C = – 20.8‰, δ^15^N = 6.4‰) are uncharacteristic of a Late Pleistocene reindeer sample. Likewise, sample UPN-115 was originally identified by macroscopic zooarchaeological analysis as red deer and not subjected to ZooMS analysis. However, its isotopic results also fall > 1.5 times outside the interquartile range of all red deer δ^13^C, and as discussed in the following paragraphs, its δ^13^C value (– 19.4‰) is uncharacteristic of a Late Pleistocene red deer sample.

Unlike the radiocarbon results, no spatial pattern is observed in the isotope data between the different excavation sectors (Supplementary Information 5). This is true both when all layers are considered together or when individual layers are considered separately. Hence, different sectors cannot be distinguished from one another based on their isotope profile, regardless of whether the sectors may represent disparate or concurrent phases on activity in the cave.

**Fig. 3:**
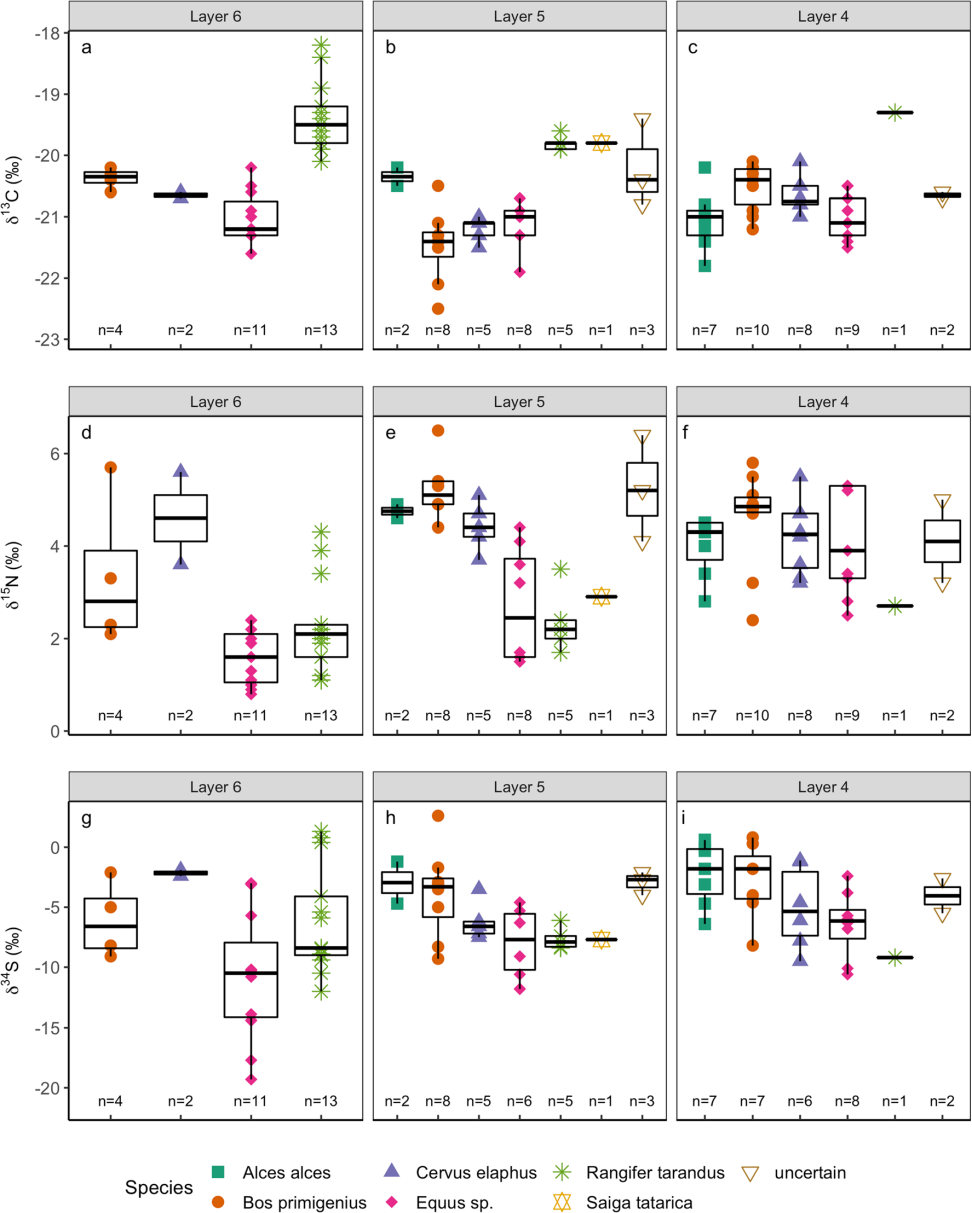
δ^13^C (top), δ^15^N (middle), and δ^34^S (bottom) bone collagen results, displayed for each layer and species. Each symbol represents one data point. Boxplots show median (central line), the 25th and 75th percentiles (bottom and top of the box), and a maximum of 1.5 times the interquartile range (whiskers)

**Fig. 4:**
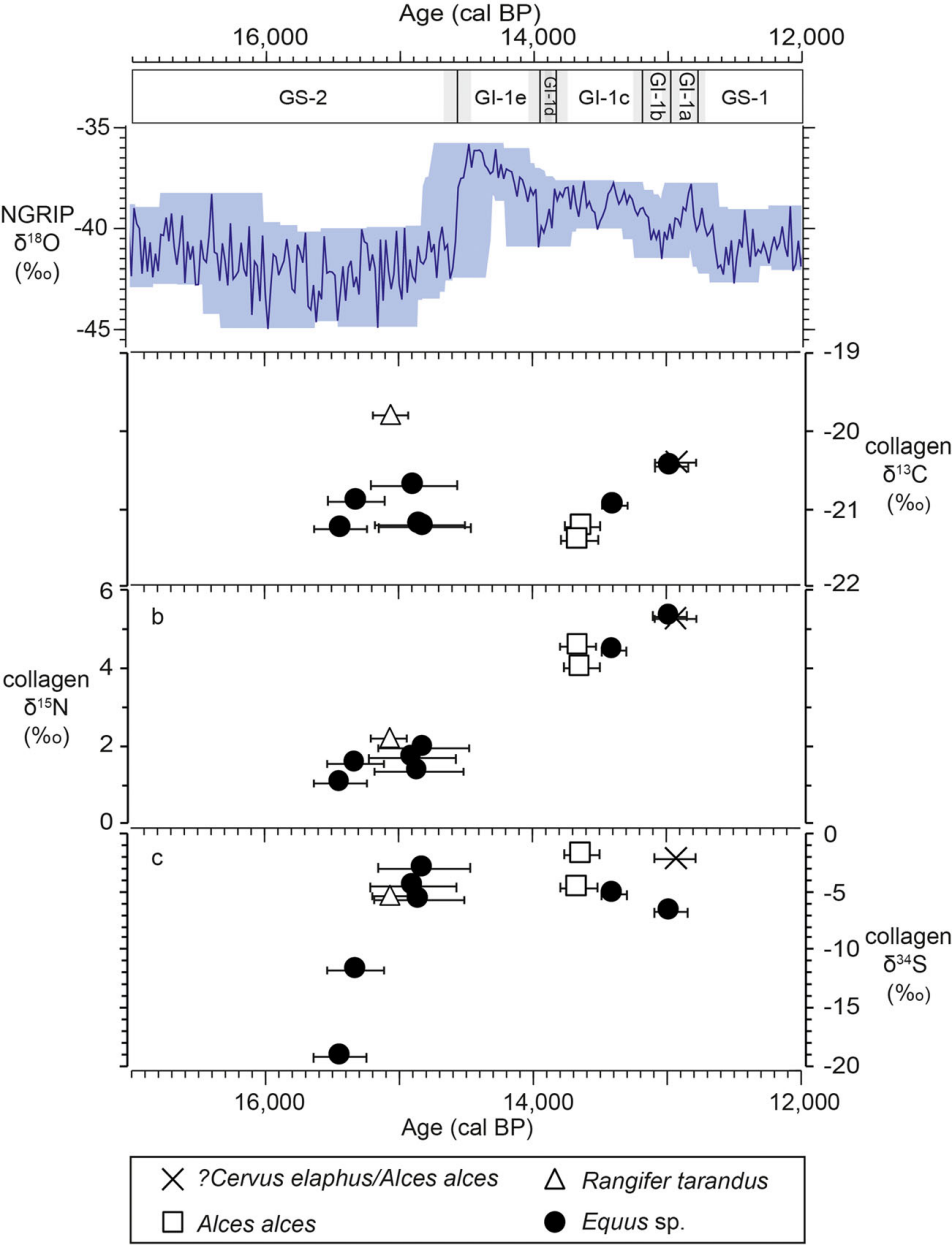
δ^13^C (**a**), δ^15^N (**b**), and δ^34^S (**c**) bone collagen results from the radiocarbon-dated samples shown alongside the NGRIP δ^18^O record with Greenland event stratigraphy shown. Age and uncertainty associated with each sample is plotted as the mid-point and range of the calibrated date at 2σ. Calibration and timescale alignments were conducted as detailed for Fig. 2

Carbon isotope values vary more strongly by species than they do by layer (Fig. 3a–c, Table 2). Reindeer and saiga have δ^13^C values ≥− 20.1‰, while horse, red deer, aurochs, and elk all have δ^13^C values ≤− 20.1‰, regardless of layer of provenance. These patterns represent the differing dietary ecologies of the species analysed. Higher δ^13^C values observed in the reindeer and saiga samples is a pattern consistently observed for these species in both modern and archaeological populations and reflects the presence of lichen in the diet (Drucker et al. 2010, Bocherens et al. 2015, Jürgensen et al. 2017; Reade et al. 2020b). Conversely, the lower δ^13^C values in horse, red deer, aurochs, and elk reflect their reliance on C_3_ vascular vegetation, which typically have δ^13^C values 2 to 4‰ lower than sympatric lichens (Brooks et al. 1997; Ben-David et al. 2001). While some inter-layer δ^13^C variation is observed (e.g. in aurochs and red deer), it is not present in all species (e.g. horse) and no clear between-layer trends are apparent (Fig. 3a–c). The lack of a temporal trend in the δ^13^C data is clearly seen when only the directly dated samples are considered (Fig. 4). This suggests that any environmentally derived variation in bone collagen δ^13^C is masked by inter-species ecological differences and intra-species background variability.

**Table 2:** Summary of bone collagen stable isotope results from each species from Layers 4, 5, and 6 at Kůlna Cave (mean ± standard deviation). Results from 5 samples where species identification is uncertain is not included in this table. All results are presented in Supplementary Information 1

Layer	Species	n	δ^15^N	δ^13^C	δ^34^S
4	*Alces alces*	7	4.0 ± 0.7	− 21.1 ± 0.5	− 2.2 ± 2.6
	*Bos primigenius*	10 (7 for δ^34^S)	4.6 ± 1.0	− 20.5 ± 0.4	− 2.8 ± 3.1
	*Cervus elaphus*	8 (6 for δ^34^S)	4.2 ± 0.8	− 20.7 ± 0.3	− 5.0 ± 3.4
	*Equus* sp.	9 (8 for δ^34^S)	4.1 ± 1.2	− 21.0 ± 0.4	− 6.5 ± 2.8
	*Rangifer tarandus*	1	2.7	− 19.3	− 9.2
5	*Alces alces*	2	4.8 ± 0.2	− 20.4 ± 0.2	− 3.0 ± 2.5
	*Bos primigenius*	8	5.2 ± 0.6	− 21.5 ± 0.6	− 3.9 ± 3.8
	*Cervus elaphus*	5	4.4 ± 0.5	− 21.2 ± 0.2	− 6.2 ± 1.6
	*Equus* sp.	8 (6 for δ^34^S)	2.7 ± 1.2	− 21.1 ± 0.4	− 8.0 ± 3.0
	*Rangifer tarandus*	5	2.4 ± 0.7	− 19.8 ± 0.1	− 7.6 ± 0.9
	*Saiga*	1	2.9	− 19.8	− 7.7
6	*Bos primigenius*	4	3.4 ± 1.7	− 20.4 ± 0.2	− 6.1 ± 3.2
	*Cervus elaphus*	2	4.6 ± 1.4	− 20.7 ± 0.1	− 2.2 ± 0.4
	*Equus* sp.	11	1.6 ± 0.6	− 21.0 ± 0.4	− 10.8 ± 5.4
	*Rangifer tarandus*	13	2.3 ± 1.0	− 19.4 ± 0.6	− 6.1 ± 4.5

In contrast, δ^15^N values display clear differences between layers (Fig. 3d–f, Table 2). Overall, δ^15^N values in Layer 6 average 2.3 ± 1.3 ‰, with 23 out of 30 samples being ≤ 2.4‰. In contrast, the Layer 4 mean δ^15^N value is 4.2 ± 0.9 ‰, with all samples (*n* = 37) being > 2.4‰ (Fig. 3d–f). The increase in δ^15^N between Layer 6 to 4 is clear when a single species is considered (e.g. horse), indicating that the between-layer difference in mean δ^15^N cannot solely be attributed to changes in the proportional representation of each species by layer. Noteworthy are the highly variable δ^15^N values from Layer 5 which encompass almost the entire range observed in the Layer 6 and 4 samples. Considering only the radiocarbon dated samples, all bone collagen δ^15^N values prior to c. 14,800 cal. BP are ≤ 2.2‰, while all those after c. 13,600 cal. BP are ≥ 4.0‰, irrespective of species attribution (Fig. 4). Low δ^15^N (< 2.5‰) is characteristic of Late Glacial herbivore bone collagen from a variety of mid and high latitude locations and has variously been linked to nutrient-poor environments, low temperatures, permafrost thaw, increased environmental moisture, or increased nutrient demand from vegetation resulting in decreased nitrogen availability (e.g. Stevens and Hedges 2004; Stevens et al. 2008; Drucker et al. 2012; Rabanus-Wallace et al. 2017; Reade et al. 2020b). Conversely, bone collagen δ^15^N > 5‰ is considered typical of environments where nitrogen supply is not a limiting factor to plant growth and environmental conditions do not inhibit the soil nutrient cycle (Drucker et al. 2012).

Notable between-layer differences in δ^34^S values are also evident. Layer 6 δ^34^S values range from − 19.3‰ to + 1.3‰, while no δ^34^S value from Layer 5 or 4 is below − 11.8‰ (Fig. 3g–h). As such, Layer 6 δ^34^S values display an approximate 50% greater range than Layers 5 and 4 combined. Considering only the radiocarbon dated samples (Fig. 4), a substantial temporal trend towards higher δ^34^S values within Layer 6 is apparent, and once reached, these higher δ^34^S values continue across the Layer 5 and 4 samples. Faunal δ^34^S values increase by c. 20‰ over a time interval of between 200 and 1000 years. This implies that the wide range of δ^34^S values in Layer 6 may represent a temporal process of change that is masked when the Layer 6 samples are considered as a homogenous dataset. The higher δ^34^S values that are represented in the Layer 5 and 4 samples occur in all species, regardless of their characteristic mobility behaviours (for example reindeer and horse are generally considered to be more mobile than aurochs and elk). This suggests that the recorded change in δ^34^S values reflects changes to the underlying environmental conditions, rather than species-specific differences in mobility and dietary behaviours.

## Discussion

### Chronology and stratigraphic relationship between the Magdalenian and Epimagdalenian

The radiocarbon dates obtained from Kůlna Cave Layers 6, 5, and 4 indicate multiple phases of Magdalenian and Epimagdalenian activity at the site. The Layer 6 phases fall within the latter millennia of GS-2.1a, which is chronologically consistent with the attribution of the faunal assemblage to Magdalenian use of the cave. Likewise, the dates from Layer 4 fall within the latter part of GI-1, which is chronologically consistent with the attribution of the faunal assemblage to Epimagdalenian use of the cave. Dates from both layers suggest multiple phases of both Magdalenian and Epimagdalenian activity. The radiocarbon results from Layer 5 do not support the presence of a temporally intermediate phase of activity between GS-2.1a and the latter part of GI-1, at least in Sectors A, C, or D of the cave. Whether people were entirely absent from Kůlna Cave between these two time periods cannot be determined with absolute certainty. However, based on currently available data, our results suggest a hiatus in the use of the cave of up to c. 1000 years, corresponding roughly to GI-1e, the warmest part of the Late Glacial Interstadial, and GI-1d, a subsequent period of generally cooler temperatures (Rasmussen et al. 2014).

**Fig. 5:**
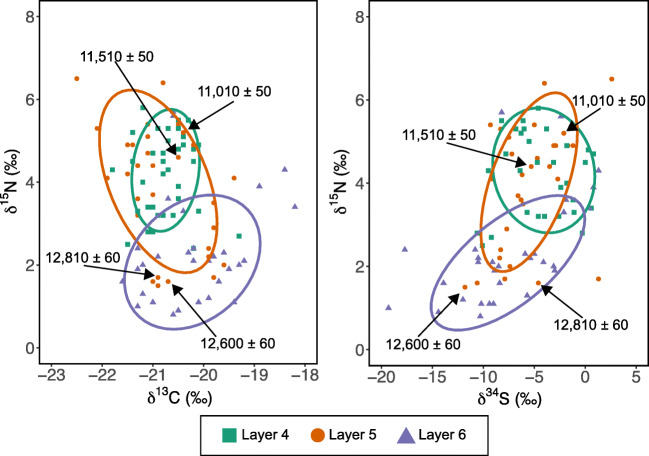
δ^13^C versus δ^15^N (left) and δ^34^S versus δ^15^N (right) scatter plot with 75% data ellipses shown for each layer. The isotope values and corresponding ^14^C BP dates for the directly data samples from Layer 5 are indicated

The interpretation of two disparate periods of activity at the site, separated by a hiatus, is supported by the stable isotope evidence, which shows clear differences between Layer 6 and Layer 4 samples (Figs. 3 and 5). The Layer 5 isotope data display a wide range of values, but when only the directly dated samples are considered, those that date to GS-2.1a fall within the isotopic ranges observed in the Layer 6 samples, while those that date to GI-1 fall within the isotopic ranges observed for Layer 4 samples (Fig. 5). The grouping of the isotopic data into two distinct clusters is further supported by hierarchical cluster analysis based on δ^15^N and δ^34^S variables (Supplementary Information 6; δ^13^C was not included in the analysis as it varies most strongly with species attribution, rather than layer). The majority of the Layer 6 (77%) and Layer 4 samples (90%) fall into different clusters (Supplementary Information 6). Although we cannot rule out the possibility of intrusive samples being included in our analysis, we suggest the weaker clustering of the Layer 6 samples compared to the Layer 4 samples is primarily due to the significant change in δ^34^S values observed within the Layer 6 data. Comparatively, the Layer 5 samples split 27% and 73% between the two groupings. We argue that the division of the Layer 5 samples into 2 clusters that are predominantly associated with the characteristic isotope values from the Layer 6 and 4 respectively (Fig. 5 and Supplementary Information 6), corroborates other lines of evidence for stratigraphic admixture. Considering these results, the Layer 5 assemblage should be treated cautiously when investigating the relationship between the Magdalenian and Epimagdalenian at Kůlna, and when inferring palaeoenvironmental information from its assemblage. While the Layer 6 and Layer 4 assemblages clearly represent palimpsests of material accumulated over multiple phases of activity at the site, we argue that they can be considered as discrete units from one another, representing different archaeological phases and (most likely) very different environmental settings.

There is only one other known site in Moravia with stratified deposits containing both Magdalenian and Epimagdalenian artefacts: Barová Cave in the central part of the Moravian Karst. Contrary to Kůlna Cave, Barová, has a very small cave entrance, which was archaeologically excavated along with the small area in front of the entrance (Seitl et al. 1986). These excavations found a limited quantity of archaeological materials that represented short term occupations related to hunting activities. Two sedimentological layers containing Magdalenian artefacts were identified: Layer 12 and 11 (Seitl et al. 1986; Maier 2015). Three Magdalenian archaeological horizons were identified within Layer 11, indicating repeat occupation of the cave (Seitl et al. 1986). The fauna associated with these horizons (reindeer, horse, white hare, polar fox, and woolly rhino) indicates a cold part of the Late Glacial, while palynological analysis suggests fluctuations between forest and steppe environments. It has been suggested that this layer correlates to the early part of the Late Glacial Interstadial (broadly equivalent to GI-1e-d) (Seitl et al. 1988). Above the Magdalenian sequence, Epimagdalenian artefacts were recognised within the sediments of Layer 10. Malacological analysis suggests a correlation of this horizon to the end of Late Glacial and the beginning of the Holocene (Seitl et al. 1986). Thus, comparing this sequence to Kůlna Cave, it seems both Magdalenian and Epimagdalenian occupation events at Barová Cave are younger than at Kůlna Cave. However, no absolute chronological data exists to confirm this. As such, it is not possible to say whether a gap existed between the Magdalenian and Epimagdalenian at Barová Cave, as seems to be the case at Kůlna Cave.

The existence of a possible interruption in occupation between the Magdalenian and Epimagdalenian in Moravia is an important question, and not one that we feel can be sufficiently addressed with the presently available data. Nevertheless, there are several reasons why we think Moravia was not depopulated between the two periods. From the point of view of both climatic and environmental conditions, there is no reason why people would abandon Moravia, especially as they appeared to persist in the region during the LGM (Nerudová et al. 2020). Thus, alternative explanations for the observed gap at Kůlna Cave need to be considered.

One possible explanation is that the gap represents a change in settlement dynamics. While there are many cave sites containing sediments dating to the period spanning GI-1 to the early Holocene, which provide abundant palaeoclimatic and environmental evidence, most are without archaeological finds (e.g. Balcarka, Výpustek and Pod Hradem). In neighbouring Bohemia, Epimagdalenian sites are situated in open-air environments (Vencl 2007) or in pseudo-karst rockshelters (Svoboda et al. 2013, 2018). If there was a greater preference for these types of locations also in Moravia, this could explain the absence of archaeological evidence dating to early GI-1 at Kůlna Cave and at other cave sites. Indeed, the difference in hunting preferences between the Magdalenian and Epimagdlenian phases at Kůlna (Valoch et al. 1969) indicates a change in the subsistence economies between the two period. It is certainly possible that the intervening period also saw different subsistence and settlement behaviours, potentially related to the development of new ecosystems and the new economic opportunities they offered.

We must also consider the generally poorer preservation of open-air sites. Overall, there are very few known Epimagdalenian sites in the Czech Republic or in adjacent regions in Poland, Slovakia, and Austria, compared to Magdalenian sites. In Poland, there is evidence for intense Magdalenian occupation, but directly dated and culturally reliable evidence for an Epimagdalenian presence is sparse, while the southeast of Poland has been suggested as a region at a periphery of the Magdalenian culture during GI-1 (Połtowicz-Bobak 2020). Yet, a continuous occupation of this area has been suggested (Bobak and Połtowicz-Bobak 2014). Therefore, it is possible that the overall limited number of known sites relates to issues of preservation and/or identification of sites attributable to this phase and thus represents a gap in our knowledge. Given this, the apparent gap observed in the archaeological record at Kůlna Cave should not be taken to represent an interruption in occupation of the wider landscape between the Magdalenian and Epimagdalenian.

### Late Glacial palaeoenvironments in the Moravian Karst

**Fig. 6:**
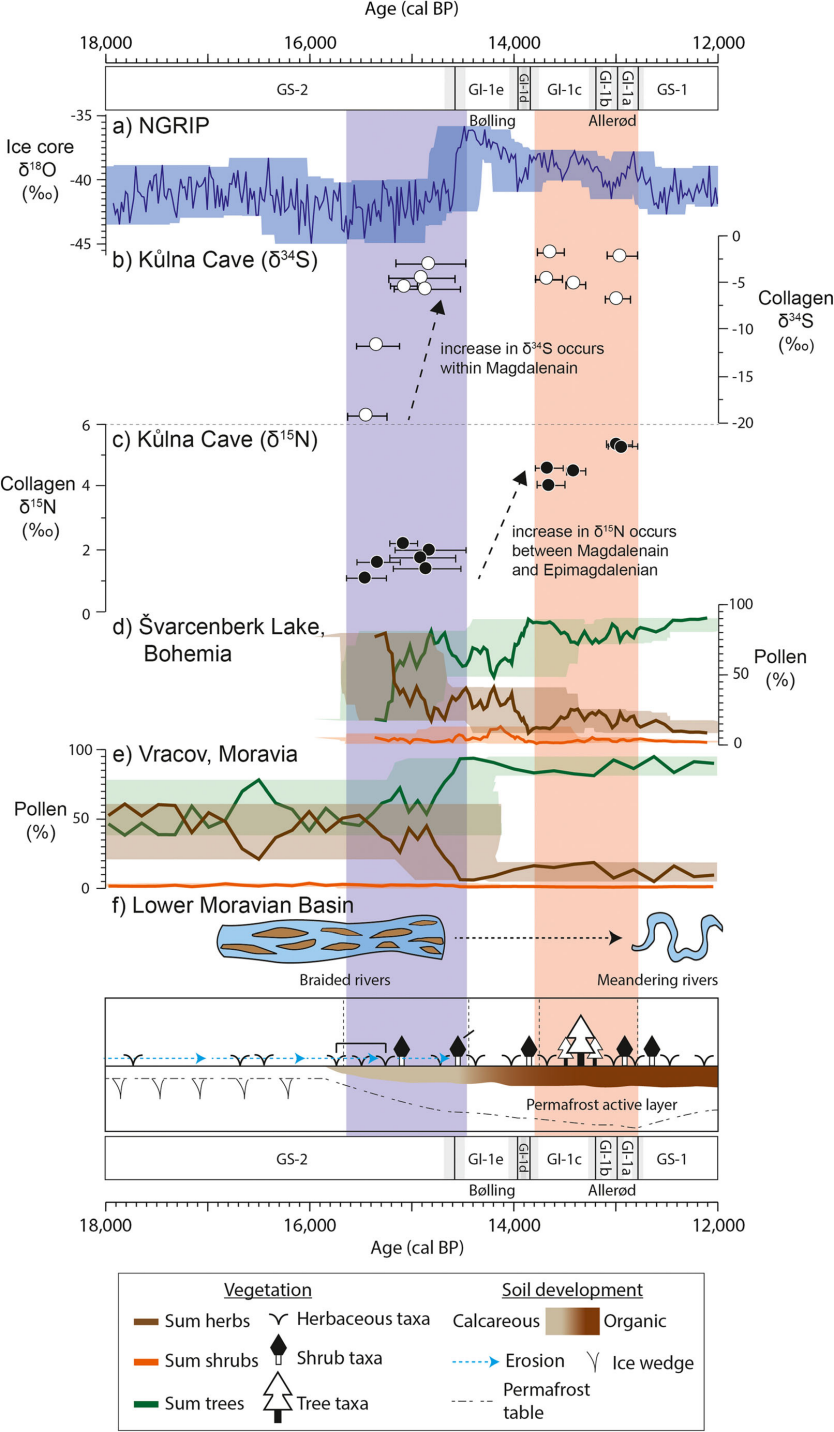
The timings of climatic and environmental changes for the past 18,000 to 12,000 years on the IntCal20 timeline, with Greenland event stratigraphy shown. Calibration and timescale alignments were conducted as detailed for Fig. 2. **a**) NGRIP δ^18^O record (Andersen et al. 2006; Svensson et al. 2006). The shaded colour represents age uncertainty at 2 sigma (roughly equated to the maximum counting errors). **b** and **c**) The sulphur (δ^34^S) and nitrogen (δ^15^N) isotope results from the dated specimens of Kulnå Cave from this study with age uncertainty of 2 sigma. **d**) Švarcenberk Lake, Bohemia (Pokorny, 2002; Hošek et al. 2014) and **e**) Vracov, Moravia (Kuneš et al. 2015; Kuneš and Abraham 2017) pollen records plotted on the age models created in this study, presented in Supplementary Information 7. The shaded colour represents the 2 sigma age uncertainty of the age model. **f**) Schematic representation of the switch of braided to meandering rivers described Kadlec et al. (2015). **g**) Schematic representation of vegetation development (plant symbols), landscape erosion (blue arrows), pedogensis (brown gradient), and permafrost depth (dash blackline) evident in palaeoenvironmental archives from the Czech Republic (Svobodová 1988; Svoboda 1994; Ložek and Cilek 1995; Pokorny 2002; Engel et al. 2010; Petr and Novák 2014; Hošek et al. 2014, 2017; Kuneš et al. 2015; Szabó et al. 2017; Kuneš and Abraham 2017)

The isotopic signatures acquired from the analysed herbivores can be taken to represent an environmental signal of the area over which the animals ranged, and in the context of our study can be used to discuss the environmental differences between the Magdalenian and Epimagdalenian at Kůlna Cave. As several different species have been analysed, the combined signal will represent a homogenised average of the various habitats that these different species utilised. Fauna from Layer 6, which is dominated by reindeer and horse, represent the time period centred upon c. 15,630 cal. BP to 14,610 cal. BP, corresponding to the final millennium of GS-2.1a (Fig. 6a). During this period, δ^15^N values are low, while δ^34^S values show a rapid increase (Fig. 6b and c). This contrasts to the Epimagdalenian fauna, in which red deer and aurochs become more common, and which center upon c. 14,140 cal. BP to 12,680 cal. BP. During this period, δ^15^N and δ^34^S values are consistently higher than the previous period (Fig. 6b and c). While both faunal δ^15^N and δ^34^S reflect the underlying soil environment, the different temporal patterns observed in the data indicate that faunal δ^15^N and δ^34^S are responding to different environmental parameters.

Faunal δ^34^S has typically been linked to animal mobility (Drucker et al. 2012, 2018; Jones et al. 2018; Wißing et al. 2019), as soil sulphur is primarily derived from mineral weathering of underlying lithology and is therefore spatially variable (Nehich 2015). However, soil δ^34^S can be strongly modified by changing bacterial reactions related to alterations in soil moisture content and oxygen status (Thode 1991; Mandernack et al. 2000). Therefore, faunal δ^34^S can also be sensitive to changing environmental conditions (Drucker et al. 2011; Reade et al. 2020a, 2020b). We argue that the temporal trend towards higher δ^34^S values evident in the directly dated Kůlna Cave Layer 6 samples (Fig. 6b) suggests that the greater variation observed in the overall Layer 6 δ^34^S data (c. 50% greater than in Layers 4 and 5 combined) most plausibly represents a change to the soil environment, rather than representing different mobility behaviours. The fact that the temporal change is clearly represented within a single species (e.g. horse; Fig. 4) supports this interpretation. Low soil δ^34^S values are produced in anaerobic environments, such as those that are water-logged (Fry et al. 1982; Bottrell and Novak 1997), and while the exact mechanisms by which this low δ^34^S sulphur becomes available to plants are not yet fully understood, low plant δ^34^S values are known to occur in these anaerobic environments, or in environments where aerobic soil conditions have recently been re-established after water-logging (Bottrell and Novák 1997; Björkvald et al. 2009; Nitsch et al. 2019). Therefore, we suggest the observed change in δ^34^S values in the Kůlna Cave data most likely indirectly record changing soil hydrological conditions between c. 15,600 and 14,900 cal. BP.

The contemporaneous δ^15^N data (Fig. 6c) does not appear to respond to the same environmental variable(s) as the δ^34^S data. The persistently low faunal δ^15^N values are characteristic of nitrogen-limited environments dominated by minerogenic soils and may reflect low environmental temperatures and/or high environmental moisture (Stevens and Hedges 2004; Stevens et al. 2008; Drucker et al. 2012; Craine et al. 2015a, b; Rabanus-Wallace et al. 2017). Low environmental temperatures are also supported by the species composition of the Kůlna Cave Layer 6 fauna, which includes cold-adapted or cold-tolerant species such as reindeer, horse, hare, bear, mammoth, arctic fox, and woolly rhino (Valoch et al. 1969; Zelinková 1998). Indeed, while gradual climatic warming did occur in the millennia following the LGM, evidence suggests the climate of Central Europe remained predominantly cold until the onset of Late Glacial Interstadial (GI-1) (Huijzer and Vandenberghe 1998; Vočadlová et al. 2015). Sedimentological archives for the region show significant landscape instability and high rates of erosion, which would have impeded the development of organic soils, while braided river systems characterised surface hydrological features (Pokorný 2002; Žák et al. 2012; Hošek et al. 2014, 2019; Kadlec et al. 2015; Fig. 6f and g). Within the Czech Republic, low pollen abundances and the prevalence of steppe and tundra taxa (*Artemisia*, Poaceae*, Helianthemum*) do not suggest a significant change in dominant vegetation type or density during this time interval (e.g Pokorný 2002; Hošek et al. 2014; Kuneš et al. 2015; Fig. 6d and e). However, a change in hydrological conditions during the latter part of GS-2.1a is evidenced by the increasing abundance of Cyperaceae pollen, a species associated with marshland environments (Pokorný 2002; Kuneš et al. 2015). It has been suggested that the development of these waterlogged environments is linked to permafrost thaw and the development of thermokarst conditions, which began around 17 ka in Central Europe, and to increased precipitation at 16–15 ka (Pokorný 2002; Žák et al. 2012; Hošek et al. 2014, 2019).

Collectively, this information provides a picture of the GS-2.1a Moravian landscape, while the faunal isotopic signature provides detail on the habitats inhabited by the prey animals of Magdalenian populations. The faunal δ^15^N and δ^34^S values indicate Magdalenian phases of activity took place in landscapes that remained dominated by minerogenic, nutrient-poor soils, but had undergone a significant increase in water availability. Drawing on palaeoenvironmental evidence from the surrounding region, we suggest the rapid increase in faunal δ^34^S values may indicate a return to well-drained soils (aerobic conditions) that followed a period of significant active thermokarst subsidence and waterlogging (anaerobic conditions). The persistence of low δ^15^N could be indicative of the unstable surface environments produced by such thermokarst processes, which would have been prone to erosion and inconducive to soil development, combined with higher precipitation amounts. This interpretation is consistent with the discussed sedimentological, geomorphological, and palynological archives from the region.

The environmental interpretations from the Layer 4 Epimagdalenian data contrast significantly to this earlier period. Consistently high faunal δ^15^N and δ^34^S values (Fig. 6b and c) suggest relatively stable environmental conditions, with the presence of more mature, nutrient-rich soils and comparatively well-developed vegetation cover (Stevens et al. 2008; Drucker et al. 2011, 2012; Reade et al. 2020b). The expansion of woodland habitat and warmer environmental conditions are also indicated by a change in species representation in the Kůlna Cave Layer 4 fauna (Valoch et al. 1969). While reindeer and horse are still present in the assemblage, their numbers decrease and more temperate species such as red deer, aruochs, and elk become more common (Valoch et al. 1969). This change is consistent with the regional pollen spectra, which document the increased presence of woodland and shrub taxa such as *Pinus*, *Betula*, *Alnus*, and *Salix* in varying proportions (Svobodová 1988; Kuneš and Abraham 2017; Fig. 6d and e). Although tundra plant species (*Artemisia* and *Poaceae*) are still present in the local pollen spectra, the disappearance of *Helianthemum* indicates the replacement of minorgenic, aeolian derived substrates, with more organic-rich soils (Kuneš and Abraham 2017). Overall, regional climate was warm and moist during this period. Chironomid inferred temperatures show summer averages of 13–14^o^C in central Poland and 14–17^o^C in western Slovakia (Płóciennik et al. 2011; Šolcová et al. 2020). Higher precipitation amounts are indicated from rising lake levels in the southern Czech Republic (Hošek et al. 2017, 2019). While in certain environmental contexts increased moisture may lead to a decrease in δ^15^N values (e.g. Rabanus-Wallace et al. 2017), an increase in precipitation when occurring alongside higher temperatures, expanding vegetation, and increased nitrogen availability would most likely result in an increase in δ^15^N values (Craine et al. 2015a, b). With the development of organic soils and increased vegetation cover, particularly woodland environments, aeolian activity and surface erosion rates decreased (Pokorný 2002; Hošek et al. 2014, 2017; Fig. 6f and g). This increased landscape stability resulted in the development of lower-energy hydrological systems, such as the meandering river systems, and increasing soil maturity, resulting in increased environmental δ^15^N values (Mol et al. 2000; Hobbie et al. 2005; Kadlec et al. 2015).

## Conclusion

This study has demonstrated that at Kůlna Cave the Magdalenian and Epimagdalenian phases of activity are likely separated by a hiatus of 400–1000 years. The Magdalenian phases appear to be restricted to the end of Greenland Stadial 2.1a and occurred within a landscape dominated by steppe and tundra herb and grass vegetation. While nutrient-poor environments appear to persist during this time period, there is evidence of significant changes to water availability. This may relate to permafrost thaw processes initiated by increasing temperatures. In contrast, the Epimagdalenian phases at Kůlna Cave appear to occur only in the latter part of Greenland Interstadial 1, in a temperate environment composed of a mosaic of comparatively mature woodland vegetation and more open grassland environments.

Kůlna Cave represents one of only two known locations in Moravia to contain both Magdalenian and Epimagdalenian assemblages in stratigraphically distinct deposits and provides by far the largest archaeological assemblages to examine the relationship between the two cultures at a single location. While there is currently no evidence that people were present at the cave in the early part of GI-1, from the viewpoint of climate and environment, there is no obvious reason why there would be a gap in occupation in Moravia at this time. As it is clear that the two cultural phases are associated with very different environmental conditions, a gap in the cave archives of the region could represent changes in settlement dynamics and/or subsistence behaviours rather than depopulation. Additional chronological investigations are certainly required to advance this discussion further.

## Supplementary Information

ESM 1 (XLSX 116 kb)

ESM 2 (PDF 826 kb)

ESM 3 (R 27.4 kb)

## Data Availability

All data generated in this study is provided in the main manuscript and supplementary data files.
