# Supplementary material for: Magdalenian and Epimagdalenian chronology and palaeoenvironments at Kůlna Cave, Moravia, Czech Republic
Source: Archaeol Anthropol Sci. 2020 Dec 17;13(1):4. doi: 10.1007/s12520-020-01254-4 (PMC7746568; doi:10.1007/s12520-020-01254-4)
Supplement: Supplementary file 2 — (PDF 826 kb) [file 12520_2020_1254_MOESM2_ESM.pdf]

SI 2: Radiocarbon corrections

Due to the pretreatment of the radiocarbon samples being undertaken at University College London's (UCL's) Institute of Archaeology Isotope Laboratory, it was necessary to apply an additional correction to the dates produced at ORAU (table S2). This was to account for the background carbon of the UCL laboratory, that was potentially introduced into the samples during pretreatment and may be different to that of the ORAU laboratory. Following the standard procedure used at ORAU and using the same two reference samples, aliquots of a cow rib from the Mary Rose shipwreck (relatively recent known aged sample) and the Latton Mammoth long bone (sample of an age beyond radiocarbon measurement) were routinely subject to parallel pretreatment at UCL along with our archaeological samples, and then analysed on the AMS at ORAU. Corrections based on results from these reference samples (Reade et al., 2020a) were calculated according to the method of Wood et al. (2010).

**Sample**	**Context**	**Species and skeletal element**	**Date number**	**Uncorrected date ^{14}C BP**	**Corrected date ^{14}C BP**
UPN-102	Layer 6, sector A, 6a	*Equus* sp., phalange	OxA-V-2775-57	12,865 ± 60	12,910 ± 60
UPN-120	Layer 6, sector A, 6g	*Equus* sp., phalange 2	OxA-V-2777-55	12,770 ± 55	12,810 ± 60
UPN-128	Layer 5, sector D, III, IV / S, depth 180-190,	*Equus* sp., metacarpal, proximal	OxA-V-2777-56	11,480 ± 50	11,510 ± 50
UPN-171	Layer 6, sector G1	*Rangifer tarandus*, humerus	OxA-V-2793-53	12,615 ± 55	12,650 ± 50

Table S2. Original and corrected ^{14}C BP values for the AMS radiocarbon dated samples reported in this study.

SI 3: Peptide mass finger-printing (ZooMS analysis)

Seven samples were analysed by peptide mass fingerprinting (ZooMS) to aid species identification. We analysed two bone samples dated by Nerudová and Neruda (2014) (OxA-25287 and OxA-25288, both from Layer 5) that had previously been unidentifiable by macroscopic zooarchaeological analysis. A further five samples were selected for ZooMS analysis where the distinction between *Cervus elaphus* (red deer) and *Rangifer tarandus* (reindeer) was uncertain. Three samples from Layer 4 (Epimagdalenian, Late Glacial Interstadial) were identified by macroscopic zooarchaeological analysis as reindeer. However, the presence of reindeer, widely considered as a cold climate indicator species, in the Czech Republic during the Late Glacial Interstadial has not been confirmed by direct dating. The only evidence for its persistence in the region after the termination of GS-2.1a comes from the un-dated remains found in Layer 4 at Kůlna Cave (Sommer et al., 2014). Two of the three Layer 4 samples thought to be reindeer produced $\delta^{13}C$ values that are uncharacteristic of the species (UPN-053 –20.6‰ and UPN-060 –20.7‰), while the third supported the initial reindeer identification (UPN-085, –19.3‰). $\delta^{13}C$ values of reindeer collagen are typically >–20‰ in Late Pleistocene samples, reflecting lichen in the diet (Drucker et al., 2010, Bocherens et al., 2015, Jürgensen et al., 2017; Reade et al., 2020b). Two samples (UPN-096 and UPN-147) from Layer 6 (Magdalenian, GS-2.1a) were identified by macroscopic zooarchaeological analysis as red deer. $\delta^{13}C$ values did not suggest either of these samples could be reindeer (–20.7‰ and –20.6‰, respectively), but as the presence of red deer is typically considered an indicator of temperate, wooded environments, these species were also subjected to ZooMS analysis to confirm the original identification.

Methodology

ZooMS pretreatment was performed in the preparative laboratory at the Oxford Radiocarbon Accelerator Unit, and prepared samples sent to the Max Planck Institute for the Science of Human History in Jena, Germany, for analysis. In all cases, collagen had already been extracted from specimens for stable isotope analysis and/or radiocarbon dating, so pretreatment for ZooMS started with prepared collagen. Pretreatment methods are based on previously published protocols by Buckley et al. (2009) and Coutu et al. (2016). Approximately 100–200 µg of dry collagen was dissolved in 100 µL of 50 mM solution of ammonium bicarbonate (CAS 1066-33-7, Fisher Scientific, 99%) in ultrapure water. A 50 µL aliquot of the sample solution was transferred to a low protein binding polypropylene microcentrifuge tube (Fisher Scientific), and 1µL of trypsin solution (Promega UK, sequencing-grade modified trypsin in acetic acid buffer) added. Samples were heated at

37°C overnight, for 12–18 h. The remainder of the collagen solution was stored at –50°C in case a second measurement was required.

After samples were removed from the oven, 10 µL of a 0.5% solution of trifluoroacetic acid (CAS 76-05-1, Fisher, HPLC grade 99+%) in ultrapure water was added to each tube to stop the digestion reaction. Solid phase extraction (SPE) was done using a 96-well SPE cartridge plate (Supelco Discovery® DSC-18) if dozens of samples were being prepared, or SPE pipette tips (Pierce™ C18 Tips) for only a few samples. In either case, the SPE phase was first conditioned with 600 µL of a solution of 0.1% TFA in a mixture of 50% acetonitrile (CAS 75-05-8, Sigma-Aldrich, ACS reagent ≥99.5%) and ultrapure water. The SPE resin was further washed with 0.1% TFA in ultrapure water. Next, 50 µL of the digested sample was loaded onto the resin, and washed with two 600 µL aliquots of 0.1% TFA in ultrapure water. The sample was eluted with 200 µL of 0.1% TFA in 50% acetonitrile:water. Samples were then left loosely capped in the fume cupboard for up to 20 h so that the solvent could evaporate.

Dry samples were re-suspended in 10 µL of 0.1% TFA, and 0.5 µL of sample was spotted onto the target plate along with 0.5 µL of a matrix solution, 10% a-cyano-4-hyroxycinnamic acid (CAS 28166-41-8, Sigma-Aldrich >99%) in ultrapure water. Plates were sent to the Max Planck Institute for the Science of Human History in Jena, Germany, for analysis on their Bruker Ultraflex II (Bruker Daltonics, Bremen) MALDI-TOF/TOF mass spectrometer. Three spectra were collected for each sample, averaged, and analysed using mMass software (Niedermeyer and Strohalm, 2012). The averaged spectra for each sample were compared to a reference database containing collagen-peptide marker masses for numerous animal genera to enable identification (Buckley et al., 2009; Welker et al., 2016).

Results and Discussion

Results from ZooMS analysis are shown in Table S3.1 and Figure S3.1. The results can be considered alongside macroscopic zooarchaeological analysis and archaeological/stratigraphical context to provide a most probable identification for each sample (Table S3.2).

Of the two samples that have been radiocarbon dated but are unidentified, the ZooMS spectra for UPN-166 (OxA-25288 12,600 ± 60 BP) identifies the sample belonging to the Equidae family, with the most probable identification being of the genus *Equus*. The ZooMS spectra for UPN-165 (OxA-25287 11,010 ± 50 BP) provides an identification to the Cervidae or Bovidae family, with the exclusion of the *Bos* and *Bison* genera (based on the F peptide) and *Rangifer* genus (based on the G peptide). Given the proportion of species belonging to the Cervidae and Bovidae family in the faunal assemblage of Layer 5, we suggest elk (*Alces alces)* or red deer is the most likely identification of this bone. Stable

isotope values for UPN-165 are 5.2‰ for $\delta^{15}N$, –20.4‰ for $\delta^{13}C$, and –2.1‰ for $\delta^{34}S$, making it isotopically indistinguishable from the range of values observed in the other analysed elk and red deer samples (Figure S3.2).

ZooMS analysis of the Layer 4 samples UPN-053 and UPN-060 rule out the possibility of either being reindeer (based on the G peptide), which corroborates the interpretation of the $\delta^{13}C$ results. ZooMS results indicate that both samples belong to either the Cervidae or Bovidae family, with the exclusion of the *Bos, Bison* and *Rangifer* genera. As with UPN-165, the most likely species in the context of Kůlna Layer 4 are red deer or elk. ZooMS analysis of the third reindeer sample for Layer 4 (UPN-085) confirmed its macroscopic zooarchaeological identification as *Rangifer*. However, without direct dating of the sample we cannot substantiate whether this demonstrates the presence of reindeer during the latter part of the Late Glacial Interstadial in Central Europe, or if it demonstrates the presence of an older, intrusive sample in the Layer 4 assemblage; both interpretations are possible.

ZooMS analysis of UPN-096 and UPN-147 from Layer 6 indicated both samples belong to either the Cervidae or Bovidae family, with the exclusion of the *Rangifer* genus. For sample UPN-096 *Bos* and *Bison* can also be excluded. While the ZooMS results do not permit the exclusion of *Bos* and *Bison* for UPN-147, this identification can be ruled out based on macroscopic zooarchaeological results; the M_3 tooth is clearly of large Cervid morphology. Based on this, we have no reason to reclassify UPN-096 and UPN-147 from their original identification as red deer, but we are unable to determine whether these samples represent red deer presence at Kůlna during GS-2.1a, or if they could represent younger, intrusive material from overlying levels. However, the presence of red deer in the Moravian Karst during GS-2.1a is not exceptional and it is found in several of the Late Pleistocene layers at Kůlna Cave (Zelinková, 1998). The presence of red deer in the Moravian Karst during colder periods of the Late Pleistocene may have been facilitated by more temperate microclimatic conditions specific to the karstic landscape, which likely offered localised pockets of woodland habitat (Nerudová et al., 2016).

**Sample**	**P1**	**A**	**A'**	**B**	**C**	**P2**	**D**	**E**	**F**	**F'**	**G**	**G'**	**ZooMS ID**
UPN-053	1105.4			1427.5		1648.6	2131.8	2792.9	2883	2899	3017.1	3033.1	Cervidae/Bovidae (excluding *Bos/Bison* and *Rangifer*)
UPN-060	1105.4			1427.5	1550.5	1648.6	2131.8	2792.9	2883	2899	3017.1	3033.1	Cervidae/Bovidae (excluding *Bos/Bison* and *Rangifer*)
UPN-085	1105.5	1150.6	1166.6	1427.7	1580.8	1648.8	2131		2883.4			3093.4	Rangifer
UPN-096	1105.5	1180.5	1196.4	1427.6	1550.6	1648.6	2131.9	2792	2883.1	2899.1	3017.1	3033.2	Cervidae/Bovidae (excluding *Bos/Bison* and *Rangifer*)
UPN-147	1105.5			1427.5			2131.8		2883.1		3017.1	3033.2	Cervidae/Bovidae (excluding *Bos/Bison* and *Rangifer*)
UPN-165	1105.5			1427.6		1648.6	2131.8	2792.9	2883.1	2899.1	3017.1	3033.1	Cervidae/Bovidae (excluding *Bos/Bison* and *Rangifer*)
UPN-166	1105.4			1427.5			2145.8	2820.9	2883.1		2983.1	2999.1	Equidae

Table S3.1 ZooMS results. Columns P1 to G’ indicate identified peaks in the mass spectra. ZooMS identification is based on these peaks

Sample number	Date code	Date	Macroscopic zooarchaeological identification	ZooMS identification	Most probable identification[1]
UPN-053	undated		*Rangifer tarandus*	Cervidae/Bovidae (excluding *Bos/Bison* and *Rangifer*)	*Alces alces/Cervus elaphus*
UPN-060	undated		*Rangifer tarandus*	Cervidae/Bovidae (excluding *Bos/Bison* and *Rangifer*)	*Alces alces/Cervus elaphus*
UPN-085	undated		*Rangifer tarandus*	Rangifer	*Rangifer tarandus*
UPN-096	undated		*Cervus elaphus*	Cervidae/Bovidae (excluding *Bos/Bison* and *Rangifer*)	*Cervus elaphus*
UPN-147	undated		*Cervus elaphus*	Cervidae/Bovidae (excluding *Bos/Bison* and *Rangifer*)	*Cervus elaphus*
UPN-165	OxA-25287	11010±50	unidentified	Cervidae/Bovidae (excluding *Bos/Bison* and *Rangifer*)	*Alces alces/Cervus elaphus*
UPN-166	OxA-25288	12600±60	unidentified	Equidae	*Equus* sp.

Table S3.2 Most probable identification based on macroscopic zooarchaeological, ZooMS and stable isotope results, and archaeological/stratigraphic context.

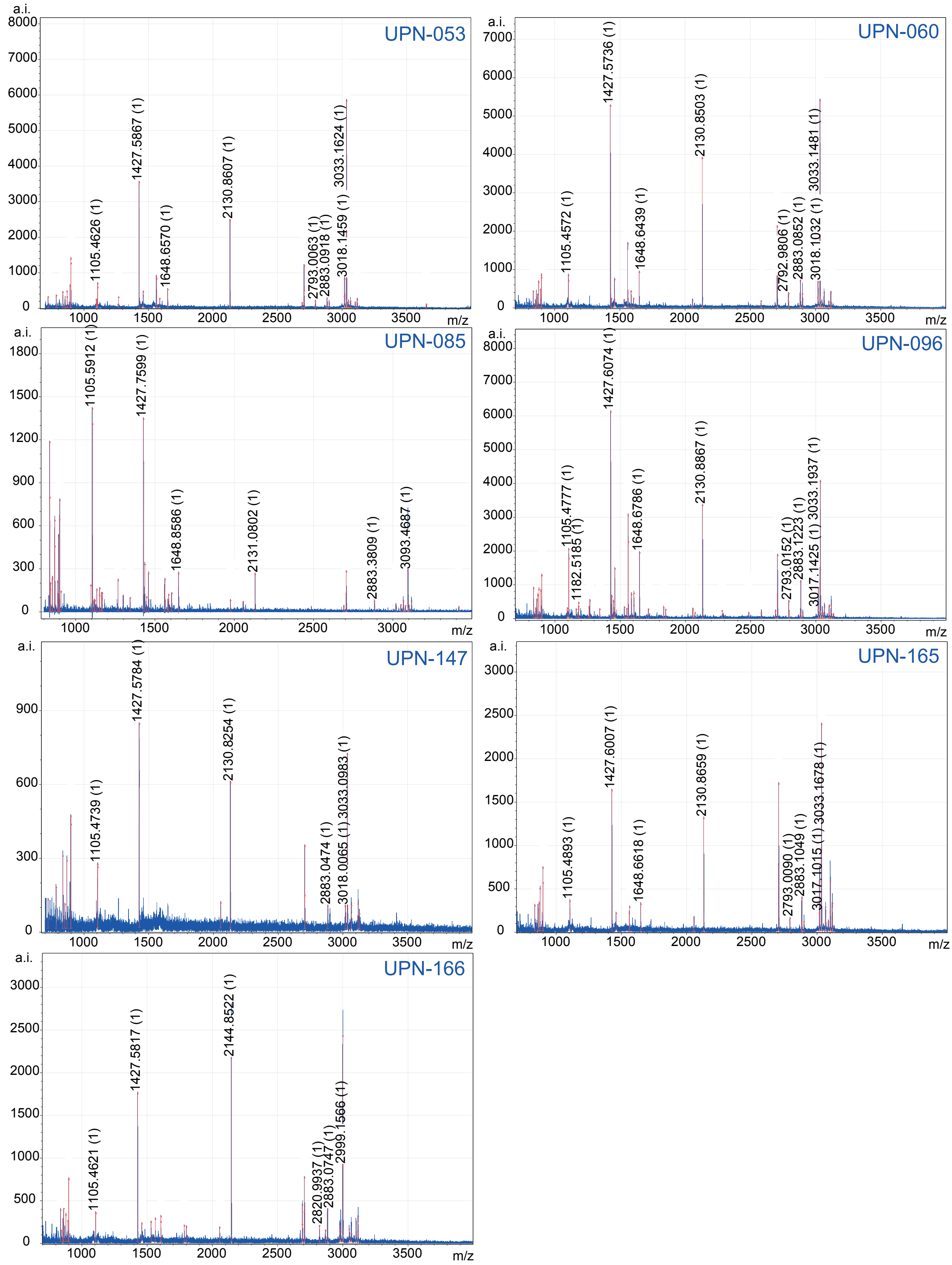

Figure S3.1 Averaged mass spectra for each sample with the peaks used for identification indicated.

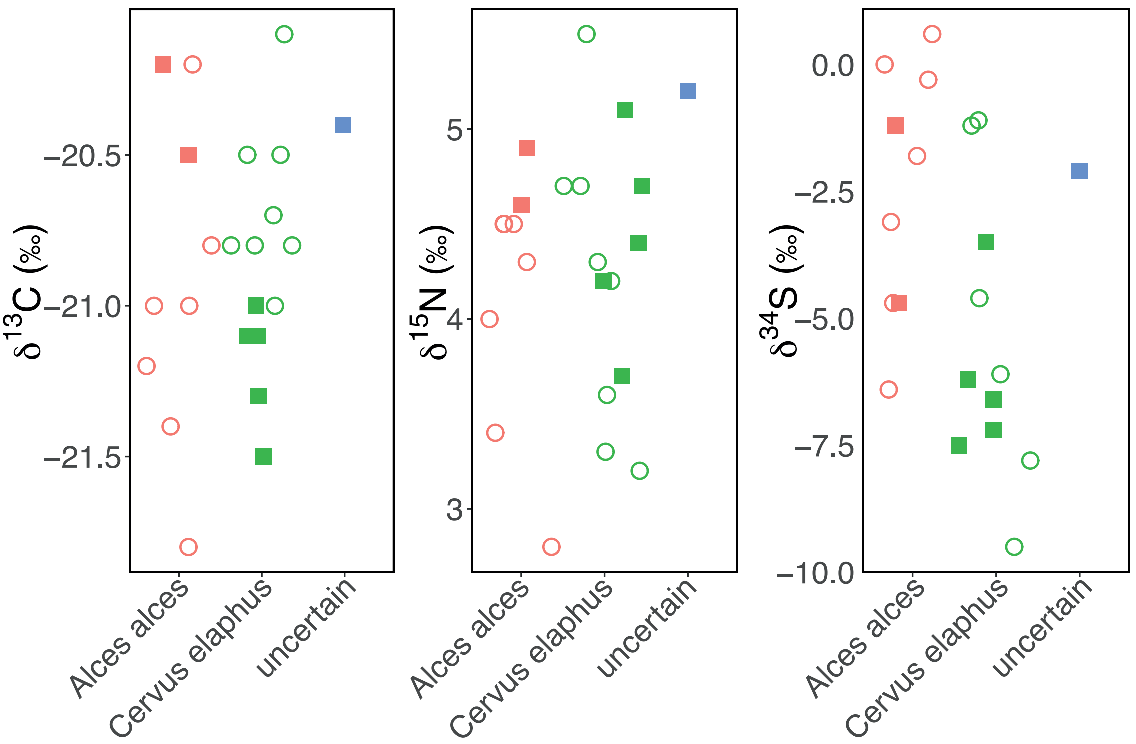

Figure S3.2 δ^{13}C (left), δ^{15}N (middle), and δ^{34}S (right) values from *Alces alces* (red) and *Cervus elaphus* (green) samples from Layer 4 (open circles) and Layer 5 (closed squares), compared to the dated sample of unknown species (UPN-165, OxA-25287, blue square)

SI 4: Bayesian age model of Kůlna Cave radiocarbon dates

The chronology of activity at Kůlna Cave was investigated using a simple phase model based on Bayes theorem and run in OxCal4.4. The limited number of radiocarbon dates available for analysis restricts the development of a more rigorous chronological model. Initially, a simple 3 phase model was tested, with the prior assumption that dates from Layers 6, 5 and 4 represented different phases of activity at the site, but that dates within each layer represent a single phase. The model showed poor agreement with these assumptions (Model Agreement Index = 0.0%), indicating with high probability that some of the radiocarbon dates included in the model were not assigned to their correct stratigraphic provenance. A second model was run using only the Layer 6 and Layer 4 data, again assuming that the two layers represented different phases of activity at the site, but that dates within each layer represent a single phase. This model showed good agreement with these assumptions (Individual Agreement Indices ranged from 86.1 – 112.0, while the Model Agreement Index was 104.1%; a value of over 60% is typically considered to represent good agreement with the prior assumptions). Based on the output from this model (table S4.1) a likely duration of activity at the cave represented in Layer 6 and 4 can be inferred.

	Modelled age (cal. BP)				
	68.2% confidence		95.4% confidence		Individual Agreement Index
	from	to	from	to	
Start Layer 6	15,625	15,270	16,055	15,117	
OxA-25290	15,093	14,890	15,175	14,590	110.2
OxA-25289	15,110	14,918	15,195	14,623	112
OxA-V-2775-57C	15,453	15,242	15,578	15,146	86.1
UPN-171	15,130	15,000	15,203	14,941	101.3
End Layer 6	15,016	14,611	15,084	14,174	
Start Layer 4	14,135	13,659	14,657	13,605	
OxA-25284	13,743	13,602	13,785	13,519	99.4
OxA-25285	13,742	13,513	13,760	13,502	100.1
OxA-25286	13,090	12,974	13,101	12,891	103
End Layer 4	13,062	12,681	13,094	11,959	

Table S4.1 Model output with agreement indices for a two-phase model using radiocarbon dates from Layers 4 and 6. Model was run in OxCal4.4 using the IntCal20 timeline, with the code given below.

OxCal code for model:

```
Plot()
 {
  Sequence()
  {
   Boundary("Start Layer 6");
   Phase("Layer 6")
   {
    R_Date("OxA-25290", 12555, 60);
    R_Date("OxA-25289", 12575, 60);
    R_Date("OxA-V-2775-57C", 12910, 60);
    R_Combine("UPN-171")
    {
     R_Date("OxA-25291", 12620, 60);
     R_Date("OxA-V-2793-53C", 12650, 50);
    };
   };
   Boundary("End Layer 6");
   Boundary("Start Layer 4");
   Phase("Layer 4")
   {
    R_Date("OxA-25284", 11820, 50);
    R_Date("OxA-25285", 11770, 55);
    R_Date("OxA-25286", 11070, 50);
   };
   Boundary("End Layer 4");
  };
 };
```

SI 5 Exploration of isotope data by excavation sector

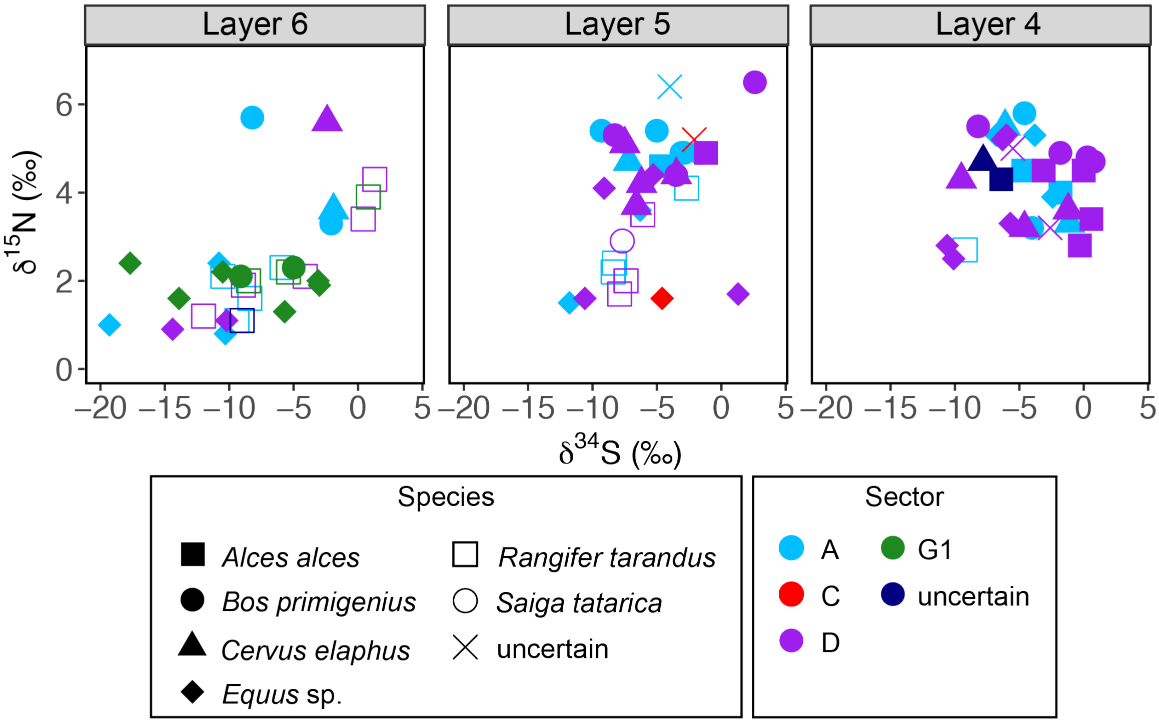

Figure S5.1 $\delta^{15}N$ versus $\delta^{34}S$ values from Layer 6 (left), Layer 5 (middle) and Layer 4 (right) samples. Colours indicate excavation sector and symbols indicate species. No relationship is observed between excavation sector and the isotopic data.

SI 6 Hierarchal cluster analysis of isotope data

Hierarchical cluster analysis was undertaken to explore grouping of samples based on $\delta^{15}N$ and $\delta^{34}S$ values. Where the C/N, C/S or N/S ratio for a sample did not fall within the range considered to indicate well preserved collagen, the sample's $\delta^{15}N$ and $\delta^{34}S$ values were excluded from the analysis (Ambrose, 1990; DeNiro, 1985; Nehlich and Richards, 2009). A hierarchical approach was favoured over a non-hierarchical approach as it makes no prior assumptions on how many groupings should exist in the data. $\delta^{15}N$ and $\delta^{34}S$ data were normalised to have a mean of 0 and standard deviation of ± 1. Divisive (top-down, DIANA) and agglomerative (bottom-up, AGNES) approaches were investigated. All approaches produced coefficients close to 1, regardless of the linkage method used, indicating strong clustering in the data (table S4.2). The agglomerative approach using Ward's minimum variance method was selected as it displayed the highest coefficient. The optimum number of clusters in the data was determined as 2 using the average silhouette width method (figure S4.2). Results of this analysis show strong clustering of Layer 4 and Layer 6 samples into distinct groups, while Layer 5 samples are more evenly distributed between the two sample groups (figure S4.3).

Method	DIANA	AGNES single	AGNES complete	AGNES average	AGNES Ward
coefficient	0.9618	0.8456	0.9622	0.9072	0.9841

Table S6.1 Coefficients of the clustering methods considered. The AGNES Ward (Ward's minimum variance) method produced a coefficient closest to 1 and was selected for subsequent analysis.

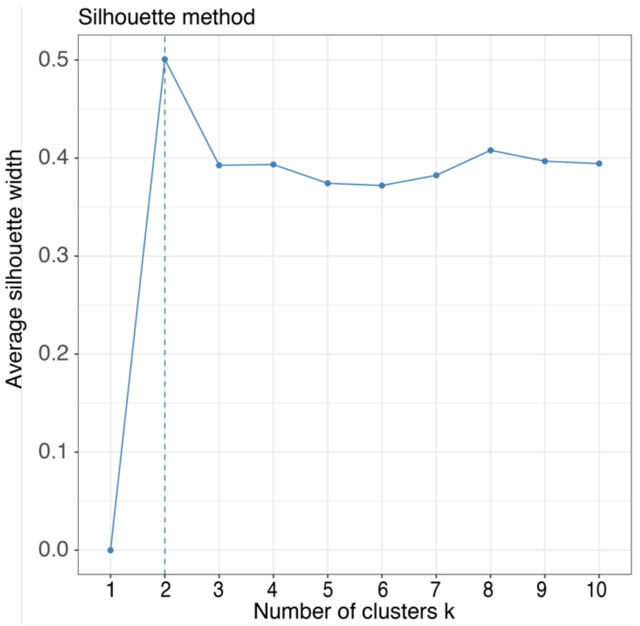

Figure S6.1 Silhouette method output, used to determine the optimum number of clusters in the data. Average silhouette width indicates 2 clusters in the data.

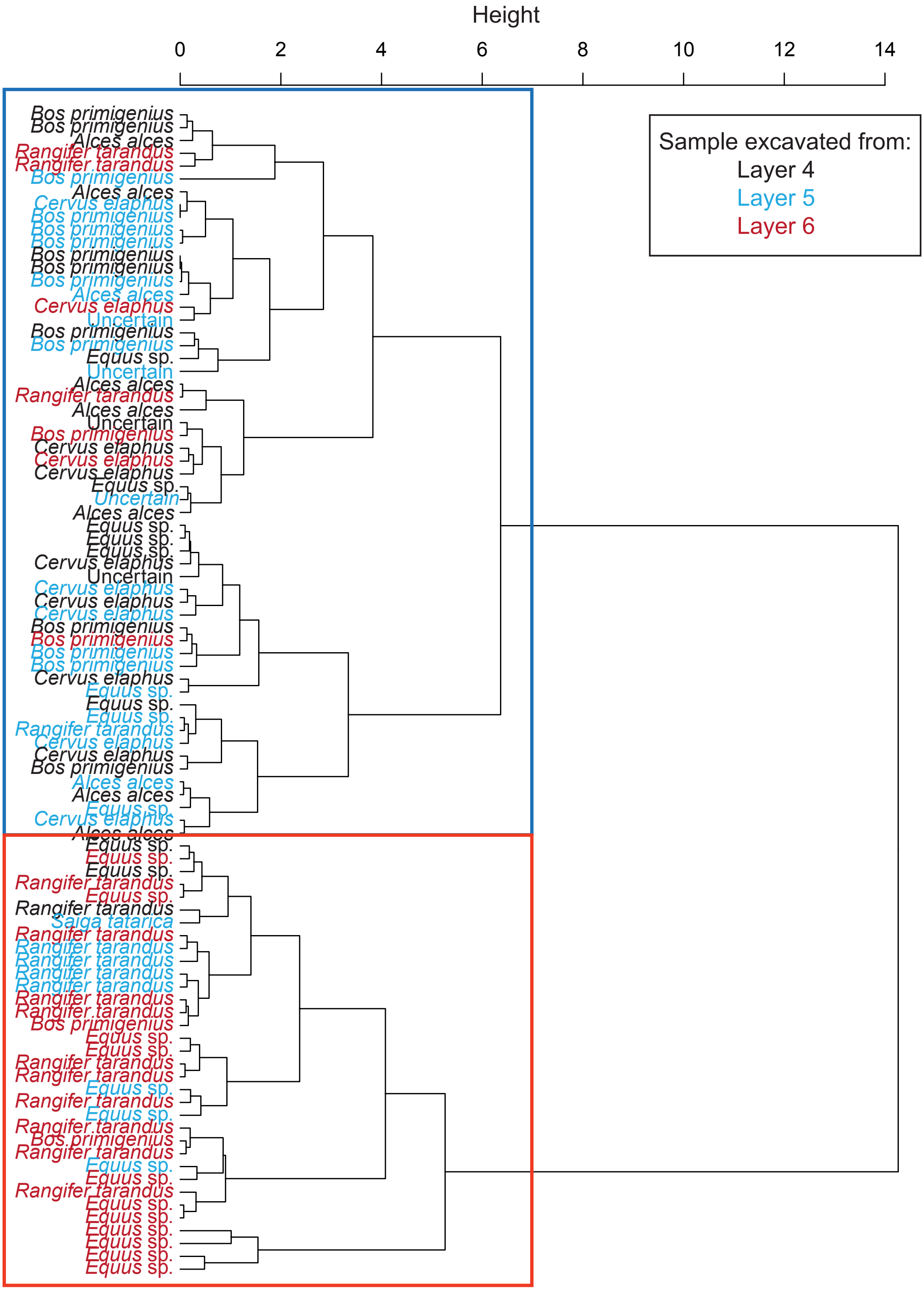

Figure S6.2 Dendrogram showing the clusters of samples based on their $\delta^{15}N$ and $\delta^{34}S$ values (clusters indicated by red and blue boxes). Each sample is labelled by its species attribution and the colour of the text indicates the layer the sample was excavated from (black = Layer 4, blue = Layer 5, red = Layer 6). The cluster analysis shows the majority of Layer 4 and Layer 6 samples falling into different clusters from one another, while the Layer 5 samples are more evenly distributed between the two clusters.

SI 7 Age models for Švarcenberk Lake and Vracov in Figure 6

Previous age model constructions for Švarcenberk and Vracov pollen records are available from the Czech Quaternary Palynological Database (Kuneš et al., 2009) and references there in, which use the IntCal13 radiocarbon calibration curve (Reimer et al., 2013). To allow accurate comparisons between the timing of regional environmental changes presented in these pollen records and that of Kůlna Cave, the ages for these pollen records were updated in this study.

Simple age models were constructed for Švarcenberk and Vracov using OxCal v4.4 (Bronk Ramsey, 2009a) and the updated IntCal20 radiocarbon calibration curve (Reimer et al., 2020). Both these new age models used the *P_Sequence* deposition model as outlined in Bronk Ramsey (2008). A model averaging approach was taken such that the programme was able to objectively derive the optimal variability in sedimentation rate based upon the radiocarbon dates themselves (Bronk Ramsey and Lee, 2013). A k value of $1.0cm^{-1}$ was used with two orders of magnitude for each site (0.01-$100cm^{-1}$). This allowed flexibility with a variable sedimentary rate within the models (Bronk Ramsey, 2008). A *General Outlier_Model* with a prior probability of 5% was applied to every radiocarbon date (Bronk Ramsey, 2009b). This results in down-weighting of dates considered to be outliers within the model rather than excluding them manually. Though, if radiocarbon dates were obviously too young or too old for the depth in the sequence, and that the model could not run, these dates were manually removed. Each record's age model had a *Boundary* applied to the base and top of the record, related to the lowest and highest core depth outlined in each site's relevant publication. For the Core top *Boundary* of Vracov, to stop the model results extending the ages into the future, the *Date* function was applied to when the core was believed to have been original recovered (Svobodová, 1992). This addition does not affect the Late Glacial ages at the base of the core, the focus of this study.

Švarcenberk Lake, Bohemia

The age model consisted of six radiocarbon dates reported in table S7.1, as published in Pokorny (2002) and Kuneš et al. (2009). *Boundaries* were applied to the depths of 'core base' and 'core top' as outlined in Pokorny (2002). The outlier analysis results showed no dates were rejected or down-weighted in the model.

Vracov, Moravia

This age model consisted of eleven radiocarbon dates seen in table S7.1. However, the three lowest radiocarbon dates (Hv-18599, Beta364949 and Poz-51954) were manually excluded from this study's age model due to these ages being consistently too young for the depth of the record resulting in the age model having problems converging and running fully.

This is in agreement with Kuneš et al. (2015) and could be linked to post-depositional reworking. The remaining radiocarbon dates were not rejected or downweighted in the resulting model from this study. Comparisons to the previous age model for Vracov by Kuneš et al. (2015) shows that this present study has older ages with larger age uncertainties. This is due to the age model of Kuneš et al. (2015) prescribing strict prior accumulation rate of 20yrs/cm compared to this study's variability in sedimentation rate, allowing for fluctuations in the deposition rate throughout the record. The choice for 20yrs/cm by Kuneš et al. (2015) may be too rigid for Late Glacial age sediments. In addition, the lack of accepted radiocarbon dates in the older part of the record resulted in older ages and uncertainty as the OxCal model extrapolated between the 'Core base' and the lowest radiocarbon date of Poz-51952. This is in addition to the different calibration curves used by this study and Kuneš et al. (2015).

Site	Sample depth (cm)	Laboratory number	Material dated	Method	Radiocarbon age (^{14}C years BP)	Used in age models?	Reference
Švarcenberk	150-153	LuA-4588	Woody stem fragments	AMS	4650 ± 100	Yes	Pokorny and Jankovska, 2000
	324-327	LuA-4589	*Trapa natans* nut	AMS	6350 ± 100	Yes	Pokorny, 2002
	390-393	LuA-4590	Woody stem fragment	AMS	9640 ± 115	Yes	Pokorny, 2002
	520-523	LuA-4591	Bulk gyttja sample	AMS	10780± 115	Yes	Pokorny, 2002
	680-683	LuA-4738	Alkali-soluable fraction from gyttja	AMS	11750 ± 120	Yes	Pokorny, 2002
	985-995	LuA-4737	*Salix* twigs	AMS	12800 ± 120	Yes	Kuneš et al., 2009
Vracov	39.5-40.5	Poz-51951	Seeds of *Carex*	AMS	670 ± 30	Yes	Kunes et al., 2015
	124.5-125.5	Poz-51947	Plant remains	AMS	1870 ± 50	Yes	Kunes et al., 2015
	184.5-185.5	Poz-51948	Plant remains	AMS	4715 ± 35	Yes	Kunes et al., 2015
	252	Beta377320	Pollen extract	AMS	9410 ± 40	Yes	Kunes et al., 2015
	280	Poz-51949	Seeds of *Naja marina*	AMS	9830 ± 60	Yes	Kunes et al., 2015
	362.5	Beta377321	Pollen extract	AMS	12390 ± 50	Yes	Kunes et al., 2015
	372	HV-1868	Bulk organic fraction	BC	12260 ± 372	Yes	Svobodová, 1992
	417	Poz-51952	Pollen extract	AMS	12890 ± 90	Yes	Kunes et al., 2015
	452	Poz-51954	Plant remains	AMS	3410 ± 120	No	Kunes et al., 2015
	480	Beta364949	Pollen extract	AMS	10880 ± 50	No	Kunes et al., 2015
	487-497	HV-18599	Bulk organic fraction containing sand	BC	10985 ± 355	No	Svobodová, 1992

Table S7.1 Radiocarbon dates and relevant information used to construct updated age models for the pollen records of Švarcenberk Lake and Vracov in this study. AMS = Accelerator mass spectrometry; BC =Beta counting.

<u>OxCal coding</u>

Švarcenberk Lake

```
Plot()
 {
  Outlier_Model("General",T(5),U(0,4),"t");
  P_Sequence("Svarcenberk",1,1,U(-2,2))
  {
   Boundary("Core base")
   {
    z=1000;
   };
   R_Date("LuA-4737",12800,120)
   {
    Outlier(0.05);
    z=990;
   };
   R_Date("LuA-4738",11750,120)
   {
    Outlier(0.05);
    z=681.5;
   };
   R_Date("LuA-4591",10780,115)
   {
    Outlier(0.05);
    z=521.5;
   };
   R_Date("LuA-4590",9640,115)
   {
    Outlier(0.05);
    z=391.5;
   };
   R_Date("LuA-4589",6350,100)
   {
    Outlier(0.05);
    z=325.5;
   };
   R_Date("LuA-4588",4650,100)
   {
    Outlier(0.05);
    z=151.5;
   };
   Boundary("Core top")
   {
    z=130;
   };
  };
 };
```

Vracov

```
Plot()
 {
  Outlier_Model("General",T(5),U(0,4),"t");
```

```
P_Sequence("Vracov",1,1,U(-2,2))
{
 Boundary("Core base")
 {
  z=495;
 };
 R_Date("Poz-51952",12890,90)
 {
  Outlier(0.05);
  z=417;
 };
 R_Date("HV-1868",12260,372)
 {
  Outlier(0.05);
  z=372;
 };
 R_Date("Beta377321",12390,50)
 {
  Outlier(0.05);
  z=362.5;
 };
 R_Date("Poz-51949",9830,60)
 {
  Outlier(0.05);
  z=280;
 };
 R_Date("Beta377320",9410,40)
 {
  Outlier(0.05);
  z=252;
 };
 R_Date("Poz-51948",4715,35)
 {
  Outlier(0.05);
  z=185;
 };
 R_Date("Poz-51947",1870,50)
 {
  Outlier(0.05);
  z=125;
 };
 R_Date("Poz-51951",670,30)
 {
  Outlier(0.05);
  z=40;
 };
 Boundary("Core top")
 {
  z=15;
 };
};
};
```

Supplementary references not in main reference list

Bronk Ramsey C (2008). Deposition models for chronological records. Quaternary Science Reviews 27(1-2): 42-60.

Bronk Ramsey C (2009a) Bayesian analysis of radiocarbon dates. Radiocarbon 51: 337-360.

Bronk Ramsey, C., (2009b) Dealing with outliers and offsets in radiocarbon dating. Radiocarbon 51: 1023-1045.

Coutu AN, Whitelaw G, le Roux P, Sealy J. (2016) Earliest Evidence for the Ivory Trade in Southern Africa: Isotopic and ZooMS Analysis of Seventh–Tenth Century ad Ivory from KwaZulu-Natal. African Archaeological Review 33: 411–435.

Kuneš P, Abraham V, Kovářík O, Kopecký M, PALYCZ contributors (2009) Czech Quaternary Palynological Database — PALYCZ: review and basic statistics of the data. Prelia 81:209–238

Niedermeyer THJ, Strohalm M (2012) mMass as a software tool for the annotation of cyclic peptide tandem mass spectra. PLoS One 7: e44913.

Sommer RS, Kalbe J, Ekström J, Benecke N, Liljegren R. (2014) Range dynamics of the reindeer in Europe during the last 25,000 years. Journal of Biogeography 41: 298-306. https://doi.org/10.1111/jbi.12193

Svobodová, H., 1992. Vývoj vegetace jizní Moravy behem pozdním glaciálu a holocénu (Vegetation development of southern Moravia during the Late Glacial and Holocene) (Diss. thesis). Institute of Botany, AS CR, Pruhonice.

Welker F., Hajdinjak M., Talamo S., Jaouen K., Dannemann M., David F., Julien M., Meyer M., Kelso J., Barnes I., Brace S., Kamminga P., Fischer R., Kessler B.M., Stewart J.R., Pääbo S., Collins M.J., Hublin J.-J. (2016) Palaeoproteomic evidence identifies archaic hominins associated with the Châtelperronian at the Grotte du Renne. Proc. Natl. Acad. Sci. U. S. A. 113: 11162–11167.

Zelinková M. (1998) Osteologický materiál z vnitřních prostor jeskyně Kůlny (Osteological material from the inside parts of the Kůlna cave). Acta Mus. Moraviae, Sci. geol., 83: 147-157.
