# Supplementary material for: Magdalenian and Epimagdalenian chronology and palaeoenvironments at Kůlna Cave, Moravia, Czech Republic
Source: Archaeol Anthropol Sci. 2020 Dec 17;13(1):4. doi: 10.1007/s12520-020-01254-4 (PMC7746568; doi:10.1007/s12520-020-01254-4)
Supplement: Supplementary file 3 — (R 27.4 kb) [file 12520_2020_1254_MOESM3_ESM.r]

| UPNORTH project sample code | Sample type | Species | Element | Layer | Sector | Unit | δ^13^C | C/N atomic ratio | Lab code | ^14^C date | Dating method | Calibrated Age BP (2σ) | Ref |
| --- | --- | --- | --- | --- | --- | --- | --- | --- | --- | --- | --- | --- | --- |
| n/a | bone | n.d | n.d | 4 | K | III-IV/F | n.d | n.d | GrN-6102 | 11,470 ± 105 | conv. | 13,162 - 13,576 | 2 |
| n/a | charcoal | n.d | n.d | 4 | B/C | I-II/L,M,P,R | n.d | n.d | GrN-11051 | 2,135 ± 45 | conv. | 1,992 - 2,305 | 2 |
| UPN-162 | bone | *A. alces* | metatarsal | 4 | A | 7f=P/VII | –21.2 | n.d | OxA-25284 | 11,820 ± 50 | AMS | 13,520 - 13,790 | 3 |
| UPN-163 | bone | *A. alces* | metatarsal | 4 | A | 1a=T/XIII | –20.7 | n.d | OxA-25285 | 11,770 ± 55 | AMS | 13,501 - 13,765 | 3 |
| UPN-164 | bone | *Equus* sp. | tibia | 4 | A | 6f=P/VIII | –19.9 | n.d | OxA-25286 | 11,070 ± 50 | AMS | 12,846 - 13,096 | 3 |
| UPN-120 | bone | *Equus* sp. | phalange 2 | 5 | A | 6g (O/8) | –21.1 | 3.4 | OxA-V-2777-55C | 12,810 ± 60 | AMS | 15,110 - 15,535 | 1 |
| UPN-128 | bone | *Equus* sp. | metacarpal | 5 | D | S/III,IV | –21.2 | 3.3 | OxA-V-2777-56C | 11,510 ± 50 | AMS | 13,298 - 13,490 | 1 |
| n/a | bone | n.d | n.d | 5 | C | I-III/K-L | n.d | n.d | GrN-6103 | 17,480 ± 155 | conv. | 20,750 - 21,733 | 2 |
| UPN-165 | bone | uncertain | unknown | 5 | C | I/L,M | –20.1 | n.d | OxA-25287 | 11,010 ± 50 | AMS | 12,783 - 13,084 | 3 |
| UPN-166 | bone | *Equus* sp. | unknown | 5 | C | I-III/K,L | –20.4 | n.d | OxA-25288 | 12,600 ± 60 | AMS | 14,565 - 15,215 | 3 |
| UPN-102 | bone | *Equus* sp. | phalange | 6 | A | 6a (T/VIII) | –21.5 | 3.3 | OxA-V-2775-57C | 12,910 ± 60 | AMS | 15,242 - 15,634 | 1 |
| UPN-171 | bone | *R. tarandus* | humerus | 6 | G1 | 38/L | –19.4 | 3.3 | OxA-V-2793-53C | 12,650 ± 50 | AMS | 14,925 - 15,254 | 1 |
| UPN-171 | bone | *R. tarandus* | humerus | 6 | G1 | 38/L | –19.4 | n.d | OxA-25291 | 12,620 ± 60 | AMS | 14,611 - 15,248 | 3 |
| n/a | charcoal | n.d | n.d | 6 | G2 | 37-38/M,O | n.d | n.d | GrN-5097 | 11,590 ± 80 | conv. | 13,305 - 13,600 | 2 |
| n/a | charcoal | n.d | n.d | 6 | D2/C | 13-14/I-L | n.d | n.d | GrN-11053 | 11,450 ± 90 | conv. | 13,166 - 13,490 | 2 |
| n/a | charcoal | n.d | n.d | 6 | D2/C | 13-14/I-L | n.d | n.d | GrN-11052 | 7,550 ± 110 | conv. | 8,048 - 8,590 | 2 |
| UPN-169 | bone | *Equus* sp. | pelvis | 6 | G1 | 37-38/M | –20.8 | n.d | OxA-25289 | 12,575 ± 60 | AMS | 14,516 - 15,179 | 3 |
| UPN-170 | bone | *Equus* sp. | tibia | 6 | G1 | 36-37/N | –20.9 | n.d | OxA-25290 | 12,555 ± 60 | AMS | 14,467 - 15,156 | 3 |

Table 1. Radiocarbon determinations from Layers 4 – 6 at Kůlna Cave. Calibrated age range is reported at 2 sigma uncertainty on the IntCal20 timeline. Reported δ^13^C isotope values were measured by IRMS as part of the AMS dating process. References: 1) this study, 2) Mook, 1988, 3) Nerudová and Neruda, 2014. n.d. = no data. Units in brackets correspond to the final system of unit division of the cave.

| **Layer** | **Species** | **n** | **δ^15^N** | **δ^13^C** | **δ^34^S** |
| --- | --- | --- | --- | --- | --- |
| 4 | *Alces alces* | 7 | 4.0 ± 0.7 | –21.1 ± 0.5 | –2.2 ± 2.6 |
|  | *Bos primigenius* | 10 (7 for δ^34^S) | 4.6 ± 1.0 | –20.5 ± 0.4 | –2.8 ± 3.1 |
|  | *Cervus elaphus* | 8 (6 for δ^34^S) | 4.2 ± 0.8 | –20.7 ± 0.3 | –5.0 ± 3.4 |
|  | *Equus* sp. | 9 (8 for δ^34^S) | 4.1 ± 1.2 | –21.0 ± 0.4 | –6.5 ± 2.8 |
|  | *Rangifer tarandus* | 1 | 2.7 | –19.3 | -9.2 |
| 5 | *Alces alces* | 2 | 4.8 ± 0.2 | –20.4 ± 0.2 | –-3.0 ± 2.5 |
|  | *Bos primigenius* | 8 | 5.2 ± 0.6 | –21.5 ± 0.6 | –3.9 ± 3.8 |
|  | *Cervus elaphus* | 5 | 4.4 ± 0.5 | –21.2 ± 0.2 | –6.2 ± 1.6 |
|  | *Equus* sp. | 8 (6 for δ^34^S) | 2.7 ± 1.2 | –21.1 ± 0.4 | –8.0 ± 3.0 |
|  | *Rangifer tarandus* | 5 | 2.4 ± 0.7 | –19.8 ± 0.1 | –7.6 ± 0.9 |
|  | *Saiga* | 1 | 2.9 | –19.8 | –7.7 |
| 6 | *Bos primigenius* | 4 | 3.4 ± 1.7 | –20.4 ± 0.2 | –6.1 ± 3.2 |
|  | *Cervus elaphus* | 2 | 4.6 ± 1.4 | –20.7 ± 0.1 | –2.2 ± 0.4 |
|  | *Equus* sp. | 11 | 1.6 ± 0.6 | –21.0 ± 0.4 | –10.8 ± 5.4 |
|  | *Rangifer tarandus* | 13 | 2.3 ± 1.0 | –19.4 ± 0.6 | –6.1 ± 4.5 |

Table 2. Summary of bone collagen stable isotope results from each species from Layers 4, 5 and 6 at Kůlna Cave (mean ± standard deviation). Results from 5 samples where species identification is uncertain are not included in this table. All results are presented in Supplementary Information 1.
